# Secretome profiling reveals acute changes in oxidative stress, brain homeostasis, and coagulation following short-duration spaceflight

**DOI:** 10.1038/s41467-024-48841-w

**Published:** 2024-06-11

**Authors:** Nadia Houerbi, JangKeun Kim, Eliah G. Overbey, Richa Batra, Annalise Schweickart, Laura Patras, Serena Lucotti, Krista A. Ryon, Deena Najjar, Cem Meydan, Namita Damle, Christopher Chin, S. Anand Narayanan, Joseph W. Guarnieri, Gabrielle Widjaja, Afshin Beheshti, Gabriel Tobias, Fanny Vatter, Jeremy Wain Hirschberg, Ashley Kleinman, Evan E. Afshin, Matthew MacKay, Qiuying Chen, Dawson Miller, Aaron S. Gajadhar, Lucy Williamson, Purvi Tandel, Qiu Yang, Jessica Chu, Ryan Benz, Asim Siddiqui, Daniel Hornburg, Steven Gross, Bader Shirah, Jan Krumsiek, Jaime Mateus, Xiao Mao, Irina Matei, Christopher E. Mason

**Affiliations:** 1 Department of Physiology and Biophysics, Weill Cornell Medicine, New York, NY USA; 2 The HRH Prince Alwaleed Bin Talal Bin Abdulaziz Alsaud Institute for Computational Biomedicine, Weill Cornell Medicine, New York, NY USA; 3 Tri-Institutional Biology and Medicine program, Weill Cornell Medicine, New York, NY 10021 USA; 4 Children’s Cancer and Blood Foundation Laboratories, Departments of Pediatrics and Cell and Developmental Biology, Drukier Institute for Children’s Health, Weill Cornell Medicine, New York, NY USA; 5 Department of Molecular Biology and Biotechnology, Center of Systems Biology, Biodiversity and Bioresources, Faculty of Biology and Geology, Babes-Bolyai University, Cluj-Napoca, Romania; 6 Department of Pharmacology, Weill Cornell Medicine, New York, NY USA; 7 Department of Nutrition & Integrative Physiology, Florida State University, Tallahassee, FL USA; 8 Center of Mitochondrial and Epigenomic Medicine, Children’s Hospital of Philadelphia, Philadelphia, PA 19104 USA; 9 Stanley Center for Psychiatric Research, Broad Institute of MIT and Harvard, Cambridge, MA USA; 10 KBR, Space Biosciences Division, NASA Ames Research Center, Moffett Field, CA USA; 11 Seer, Inc., Redwood City, CA 94065 USA; 12 Department of Neuroscience, King Faisal Specialist Hospital & Research Centre, Jeddah, Saudi Arabia; 13 Space Exploration Technologies Corporation (SpaceX), Hawthorne, CA USA; 14 Department of Basic Sciences, Division of Biomedical Engineering Sciences (BMES), Loma Linda University Health, Loma Linda, CA 92350 USA; 15 Meyer Cancer Center, Weill Cornell Medicine, New York, NY 10065 USA; 16 The Feil Family Brain and Mind Research Institute, Weill Cornell Medicine, New York, NY 10021 USA; 17 WorldQuant Initiative for Quantitative Prediction, Weill Cornell Medicine, New York, NY 10021 USA

**Keywords:** Epigenomics, Metabolomics

## Abstract

As spaceflight becomes more common with commercial crews, blood-based measures of crew health can guide both astronaut biomedicine and countermeasures. By profiling plasma proteins, metabolites, and extracellular vesicles/particles (EVPs) from the SpaceX Inspiration4 crew, we generated “spaceflight secretome profiles,” which showed significant differences in coagulation, oxidative stress, and brain-enriched proteins. While >93% of differentially abundant proteins (DAPs) in vesicles and metabolites recovered within six months, the majority (73%) of plasma DAPs were still perturbed post-flight. Moreover, these proteomic alterations correlated better with peripheral blood mononuclear cells than whole blood, suggesting that immune cells contribute more DAPs than erythrocytes. Finally, to discern possible mechanisms leading to brain-enriched protein detection and blood-brain barrier (BBB) disruption, we examined protein changes in dissected brains of spaceflight mice, which showed increases in PECAM-1, a marker of BBB integrity. These data highlight how even short-duration spaceflight can disrupt human and murine physiology and identify spaceflight biomarkers that can guide countermeasure development.

## Abstract (web summary)

Here the authors report spaceflight secretome profiles by integrating plasma proteome, metabolome, and extracellular vesicles/particles proteome from the SpaceX Inspiration4 crew, which showed differences in coagulation, oxidative stress, and brain-enriched proteins.

## Introduction

As spaceflight and long-term human missions become more attainable, minimally invasive approaches to monitor physiological responses to spaceflight in diverse populations will be key to preventing acute and long-term complications^1^. The plasma proteome provides a catalog of proteins circulating in the blood, and thus it can paint an informative picture of the systemic physiological state of astronauts^2^, yet there are scant studies of astronaut plasma proteomics. Previous plasma proteomic studies of long-duration spaceflight reported changes in platelet function, coagulation, hemostasis, immune function, and metabolism^3–5^. In addition, various ground-based analog studies (i.e. head-down bed rest) have reported blood proteomic changes including complement activation, acute inflammatory responses, fibrinolysis, and thrombosis^6,7^. Moreover, da Silveira et al. demonstrated that mitochondrial disruption and oxidative stress were the key hubs for increased health risks^7^, based on miRNA biomarkers in plasma.

Plasma-circulating extracellular vesicles and particles (EVPs) and metabolites provide additional information regarding the cellular phenotype and state of distal organs and tissues^8,9^. EVPs are actively released into the peripheral circulation at concentrations of > 10^9^ vesicles/mL, and are demonstrated biomarkers in cancer^8^, traumatic brain injury^10^, and autoimmune diseases^11^. Thus, EVP cargo analysis could be used to monitor health risks associated with space exploration, such as spaceflight-associated neuro-ocular syndrome (SANS)^12^ and thrombosis^13,14^, but studies of spaceflight-induced changes in EVP cargo have been limited^15^. Moreover, for metabolites, prior missions have focused on markers of bone metabolism and musculoskeletal deconditioning, including increased osteogenesis and resorption markers (e.g., osteocalcin, sclerostin, parathyroid hormone, osteoprotegerin, and RANKL) in the blood of crew members^16–19^, and could benefit from a wider profile. The decreased muscular activity associated with spaceflight leads to a loss of nitrogen and an inability to maintain whole-body protein synthesis rates, exacerbated by hypocaloric intake^20^, but this has not been examined in EVPs or metabolites in recent missions. Prior missions have also revealed spaceflight anemia, with circulating red blood cells and plasma volume decreases of 10–15%, leading to increased iron availability^21^, though the effect of these changes on EVP cargo is unknown.

The NASA Twins study provided the first profile and multi-omic analysis of the plasma proteome, EVP proteome, and plasma metabolome, namely from a one-year, long-duration mission^22^. For that study, EVP protein cargo showed changes related to physiological stress, systemic inflammation, and the first indication of brain-derived proteins in plasma-circulating EVPs^15^. However, the NASA Twins study only used untargeted plasma proteomics (capturing 292 proteins) to discern an increased ratio of apolipoprotein B (APOB) to apolipoprotein A1 (APO1)^22^. Of the 60 plasma metabolites involved in the tricarboxylic acid (TCA) cycle, glycolysis, amino acid, fatty acid, ketone body, and pyrimidine metabolism, no significant changes for in-flight levels were found^22^. However, it is necessary to expand the NASA Twins study findings to an increased number of samples, larger coverage of metabolomics, broader coverage of plasma proteomics, longitudinal data of EVP proteomics, and a systematic integration and meta-analysis of secretome changes with other multi-omic data^7^.

The SpaceX inspiration4 (i4) mission provided the first opportunity for such an expanded study, and it also was notable as the first all-civilian space flight mission, featuring 2 male and 2 female astronauts aged 29 to 50 years, who traveled into space for 3 days in orbit at 590 km altitude. We profiled the spaceflight secretome (plasma proteome and metabolome, and EVP proteome) of the i4 astronauts at three pre-flight (L-92, L-44, L-3) and three post-flight timepoints (R + 1, R + 45, R + 82). We performed differential and pathway enrichment analysis of the proteome and metabolome to reveal the biological landscape of the secretome changes induced by 3-day spaceflight. Then, we delineated the contribution of blood and immune cells by comparing secretome and transcriptomic profiles obtained from the i4 single-nuclei PBMC and whole-blood RNA-seq. We performed thiobarbituric acid reactive substance assay (TBARS) measurement assays, EVP immune cell marker profiling, Western blots, and ELISA to validate findings. The unique insight of integrating these different modalities (proteomics, metabolomics, and transcriptomics) in our study provides the largest comprehensive assessment of the systemic physiologic and secreted changes resulting from spaceflight exposure to date.

## Results

### Changes in the proteomic profile of plasma and EVPs after 3-day spaceflight

**Fig. 1:**
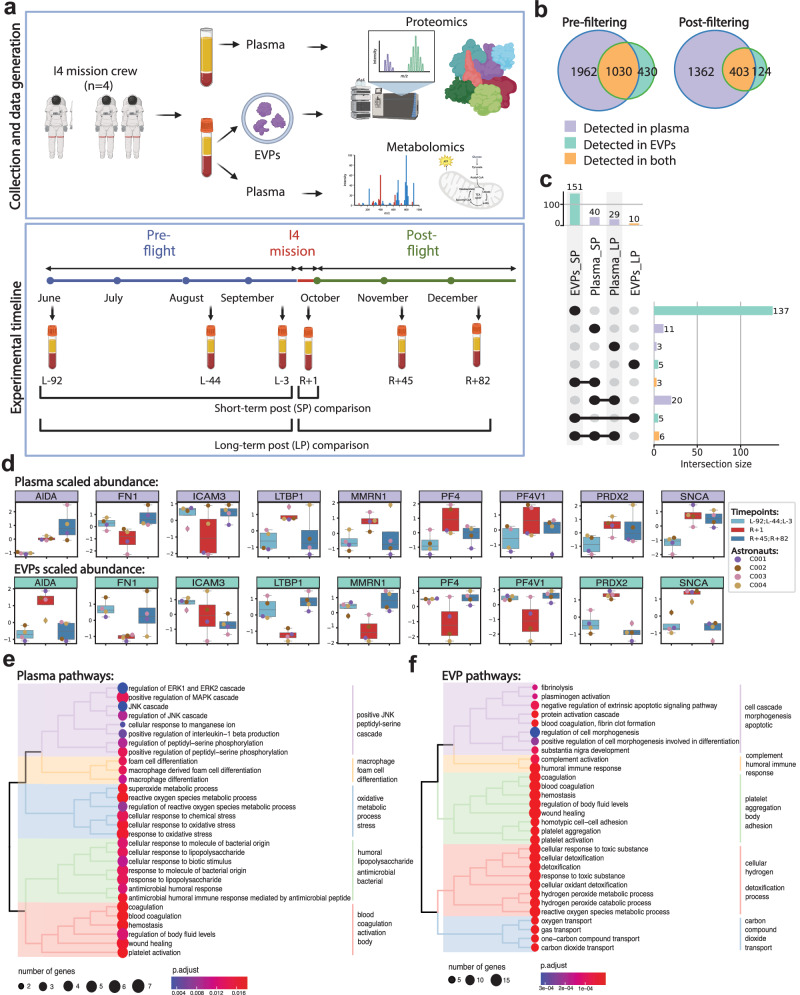
Changes in the proteomic profile of plasma and EVPs after 3-day spaceflight. **a** Overview of study design, sample collection, and processing of plasma and EVP proteomics. **b** Venn diagram of proteins measured in plasma and EVP, before (left) and after (right) filtering, based on the coefficient of variance, low abundance, and number of not assessed (NAs). **c** Upset plot showing the overlap of differentially abundant proteins (adjusted *p*-value < 0.05, |logFC | >1) across the different comparisons performed in plasma and EVPs. Differential abundance analysis was performed with *limma* with the following model ~astronaut+flightSatus and p-values have been adjusted to control the false discovery rate **d** Boxplots of the scaled abundance of the 9 proteins differentially abundant in both plasma and EVPs. Where available, data represents *n* = 4 astronauts averaged at the indicated condition (preflight and long-term postflight). Plasma data is the average of two technical replicates, EVP data represents one technical replicate per astronaut and timepoint. Boxes show the quartiles of the dataset while the whiskers extend to show the rest of the distribution except for outliers. **e** Gene Ontology enrichment was performed using clusterProfiler::enrichGO() on differentially abundant proteins in plasma (adjusted *p*-value < 0.05, |logFC | >1) at R + 1 vs. Preflight. Biological processes (BP) were selected, and *treeplot* was used to organize significant pathways (adjusted p-value < 0.05) into biologically relevant clusters. **f** Gene Ontology enrichment was performed using clusterProfiler::enrichGO() on differentially abundant proteins in the EVPs (adjusted *p*-value < 0.05, |logFC | >1) at R + 1 vs. Preflight. Biological processes were selected, and *treeplot* was used to organize and cluster the significant pathways (adjusted *p*-value < 0.05) into biologically relevant clusters. Source data are provided as a Source Data file.

To gain insight into secretome changes after 3 days of spaceflight, we profiled the plasma EVP proteins and plasma metabolites of the four i4 mission crew members (Fig. 1a) at three pre-launch dates (L-92, L-44, and L-3) and three post-flight timepoints upon return to Earth (R + 1, R + 45, R + 82) (Fig. 1a). For plasma proteomics, plasma was isolated using Cell Preparation Tubes (CPT) and processed with Seer’s 5-nanoparticle Proteograph assay^23^, while plasma EVPs were isolated as previously described^24,25^, and proteins were analyzed by nano-LC-MS/MS (Fig. 1a). Plasma metabolites were extracted using Aqueous Neutral Phase (ANP) hydrophilic and C18 hydrophobic liquid chromatography, and metabolites were identified and quantified by MS (Fig. 1a). We identified a total of 2,992 unique plasma proteins and 1,443 unique EVP proteins, with an overlap of 1,030 proteins shared by plasma and EVPs (Fig. 1b). These shared proteins are likely plasma EVP proteins as well as proteins that can be either soluble/free or EVP-associated. Plasma and EVP proteins were then filtered based on the number of not-detected (NAs) so that at least one condition has no missing data and coefficient of variation (<0.5), with 1,765 plasma circulating proteins remaining in plasma and 527 in EVPs (Fig. 1b).

To profile acute and long-term changes in the secretome after 3-days of spaceflight, we performed two comparisons: 1) immediately post-flight (R + 1) vs. all pre-flight (L-92, L-44, L-3) timepoints, as a measure of acute changes (short-term postflight, or SP) and 2) all post-flight (R + 1, R + 45, R + 82) vs. all pre-flight (L-92, L-44, L-3) timepoints, representing long-term changes (long-term postflight, or LP). Interestingly, even though fewer unique proteins were detected in EVPs, we identified more differentially abundant proteins (DAPs) in EVPs compared to plasma (151 DAPs in EVPs vs. 40 DAPs in plasma) at R + 1 (Fig. 1c, Supplementary Fig. 1). Importantly, the majority of EVP DAPs returned to pre-flight levels over time, with only 10 EVP DAPs (6.62%) remaining differentially abundant long-term post-flight (Fig. 1c). However, most plasma DAPs (72.5%) remained differentially abundant at the last timepoint (R + 82) (Fig. 1c), indicating a greater degree of recovery, and longer duration, than the EVP DAPs.

In addition, the EVP and plasma proteomes provided distinct information about spaceflight-associated changes. Specifically, 9 DAPs were shared between EVPs (5.96%) and plasma (22.5%) immediately post-flight (Fig. 1c). The 9 overlapping DAPs were Platelet factor 4 (PF4), Latent-transforming growth factor beta-binding protein 1 (LTBP1), Platelet factor 4 variant 1 (PF4V1), Alpha-synuclein (SNCA), Peroxiredoxin 2 (PRDX2), Fibronectin 1 (FN1), Axin interactor, dorsalization associated (AIDA), Multimerin 1 (MMRN1), and Intercellular adhesion molecule 3 (ICAM3) (Fig. 1d). Of these, PRDX2 and SNCA increased, while ICAM3 decreased in both EVPs and plasma. Since PRDX2 is an antioxidant enzyme^26^, its elevation in EVPs and plasma at R + 1 may indicate elevated oxidative stress. Increased SNCA level in the blood, including in EVPs, is a potential indicator of brain inflammation and stress^27,28^. Also of note, ICAM3 downregulation may reflect impairments in T cell-Dendritic cell (T-DC) interactions and immune function, as ICAM3 is crucial for the initial interaction between these two immune cells^29^. In addition, proteins associated with wound healing and coagulation, including PF4, PF4V1, and LTBP1, were increased in the plasma, but decreased in EVPs, potentially as a consequence of EVP capture in clots associated with spaceflight-induced thrombosis (Fig. 1d and Supplrmentary Fig. 1)^14,15^. However, PF4 and PF4V1 levels quickly returned to baseline, suggesting that the pro-thrombotic effect of spaceflight is temporary and reversible; these shifts of PF4 in plasma and EVPs were confirmed by ELISA (Supplementary Fig. 2a, b).

To gain a functional understanding of plasma and EVP proteome changes after spaceflight, we performed biological pathway enrichment analysis for the R + 1 DAPs in both plasma and EVPs. Interestingly, though individual DAPs mostly differed in plasma relative to EVPs, pathways enriched in these DAPs showed a consistent profile in plasma and EVPs. DAPs involved in reactive oxygen species (ROS) production, oxidative stress, wound healing, coagulation, immune function, and hemostasis pathways were enriched in both plasma and EVP profiles (Fig. 1e, f). These findings indicate that the plasma secretome reflects the hematologic changes (hemostasis, wound healing, coagulation), immune response/inflammation changes, and molecular changes in ROS metabolism after the 3-day spaceflight. We also note the increased abundance of several proteins related to the complement pathway, such as FCN3 which remains upregulated even at R + 45. This was validated in EVPs by western blotting (Supplementary Fig. 2c).

### Changes in the metabolic profile of plasma after 3-day spaceflight

**Fig. 2:**
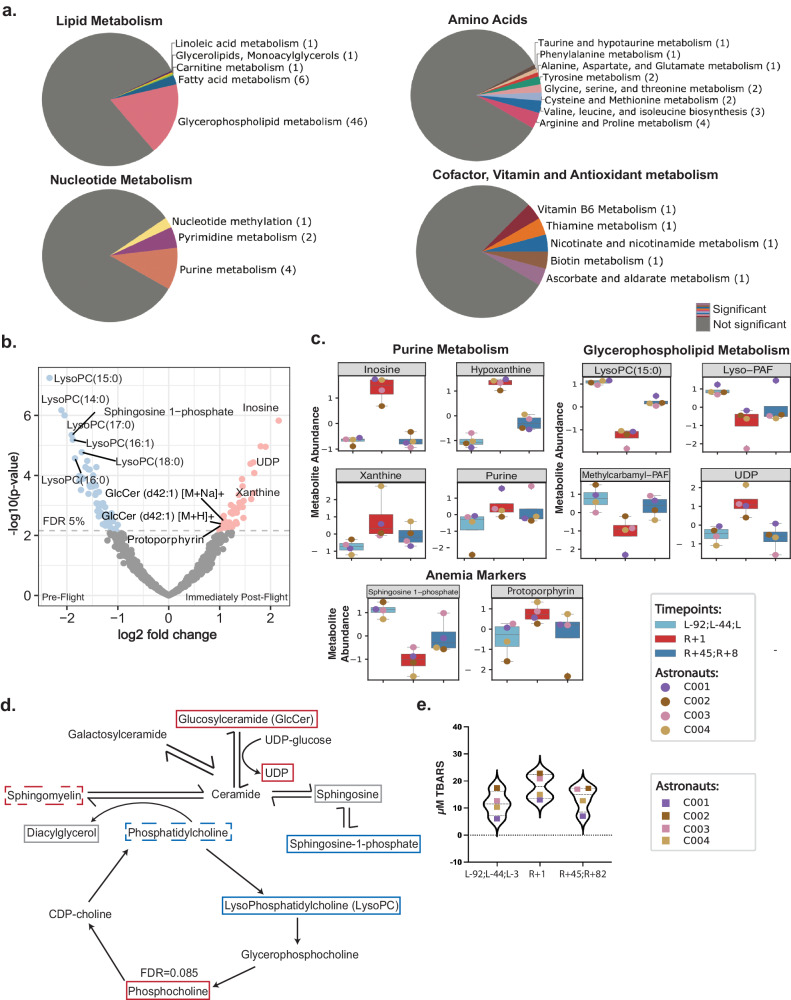
Changes in plasma metabolites after 3-day spaceflight. **a** Pie charts showing pathway annotations of differentially abundant metabolites (adjusted p-value < 0.05, |logFC | >1) at R + 1 vs. Preflight. Gray portions of the pie chart represent measured but insignificant metabolites in the specified category, while colored portions are labeled by metabolite pathway name and frequency. **b** Volcano plot of metabolites based on differential abundance (logFC>1 in red, logFC < −1 in blue) at R + 1 vs. Preflight. Labeled points are differentially abundant metabolites with connections to spaceflight-related anemia, inflammation, and oxidative stress. **c** Box plots of the scaled abundance of select differentially abundant metabolites associated with purine metabolism, glycerophospholipid metabolism, and anemia and hemolysis. Data represents *n* = 4 astronauts averaged at the indicated condition with one technical replicate per astronaut at each timepoint. Boxes show the quartiles of the dataset while the whiskers extend to show the rest of the distribution except for outliers. **d** Diagram of the sphingomyelin cycle, which is enriched in spaceflight-affected metabolites. Annotation boxes represent metabolites significantly changed at R + 1 vs. Preflight, with increased abundance indicated in red, decreased abundance in blue, and no significant change in gray (only boxed metabolites were measured). The dashed boxes indicate significant changes in some, but not all, chain lengths of the indicated lipid species. **e** Violin plots showing the results of Thiobarbituric acid reactive substances (TBARS) assay performed on astronaut plasma. Repeated measures one-way ANOVA was performed to assess significance. Source data are provided as a Source Data file.

To capture spaceflight-related metabolic changes, we next profiled 1,135 metabolites in the plasma of the i4 crew using ANP hydrophilic and C18 hydrophobic liquid chromatography coupled with mass spectrometry^30^. Differential analyses of the metabolomics data identified a variety of metabolic pathways affected by spaceflight, with 100 differentially abundant metabolites (DAMs) identified when comparing the pre-flight (L-92, L-44, L-3) to immediately post-flight (R + 1) timepoint (Fig. 2a, b). Notably, none of these DAMs remained differentially abundant at timepoints after R + 1, indicating that these metabolic changes are acute, and also that metabolic homeostasis is restored rapidly upon return to Earth.

Of the metabolites affected immediately post-flight, those involved in the purine metabolism pathway showed systematically increased abundance. Inosine, the metabolite with the largest positive fold-change (Fig. 2b), together with its precursor metabolite, purine, and its post-degradation metabolites, xanthine, and hypoxanthine, are all components of purine metabolism (Fig. 2c). We found that many of the most significantly altered metabolites belonged to the glycerophospholipid metabolism pathway (Fig. 2a), likely driven by a decreased abundance of lysophospholipid (LysoPC) and phosphatidylcholine (PC) species such as methylcarbamoyl platelet-activating factor (PAF) C-16 and LysoPAF C-16 (Fig. 2b, c)^31^. Accompanying these changes, many metabolites involved in the sphingomyelin cycle were differentially abundant, including an increase in uridine diphosphate (UDP), some sphingomyelin and glucosylceramides (GlcCer) species, and a decrease in sphingosine-1-phosphate (S1P) (Fig. 2c, d).

Since phosphatidylcholines (PCs) are the most abundant phospholipid species in cellular membranes, and membrane stability is disrupted by lipid peroxidation, we hypothesized that the decrease in PCs and LysoPCs (Fig. 2d) was indicative of lipid peroxidation secondary to spaceflight-induced production of free radicals^32,33^. The lipid peroxidation cascade generates a number of different intermediates depending on the target lipid species^34^. Malondialdehyde (MDA) is one of the major byproducts of lipid peroxidation and widely used as a biomarker of oxidative stress^34^. Therefore, to measure the overall lipid peroxidation in plasma, we quantified the levels of MDA adducts with thiobarbituric acid (TBA) using the thiobarbituric acid reactive substance assay (TBARS assay) (Fig. 2e), which revealed that lipid peroxidation was significantly increased (*p*-value = 0.0013) immediately post-flight (R + 1) and returned to baseline levels after several weeks (R + 45).

### Upregulation in production of antioxidants in the plasma and EVPs in response to spaceflight

**Fig. 3:**
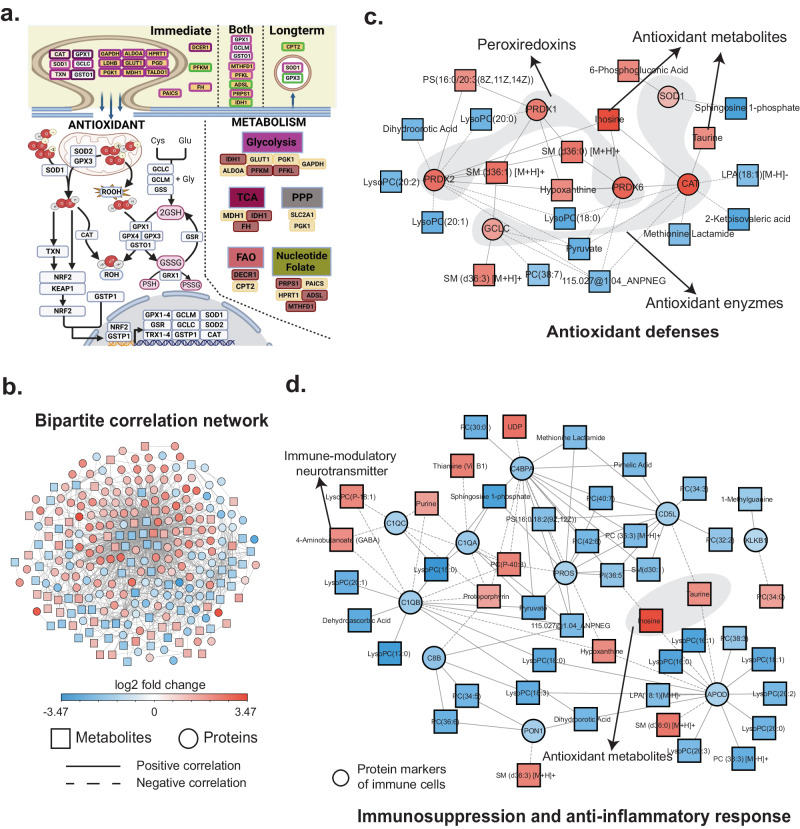
Integrated proteomic and metabolomic analyses reveal a common signature of antioxidant defense and immune dysfunction. **a** The top (plasma) indicates significantly differentially abundant proteins in the plasma and EVPs from immediate or long-term post-flight or both immediate and long-term post-flight groups compared to ground controls. Antioxidant proteins are white-colored, and proteins involved in mitochondrial metabolism are orange. The purple edges represent upregulated proteins, and the green edges represent downregulated proteins. The lower panel (inside the cell) shows the antioxidant and mitochondrial metabolism protein functions within the cell. **b** Overview of the bipartite correlation network with proteins and metabolites as nodes. Ellipses depict proteins, and metabolites are represented by square nodes. The edges indicate significant correlations between the nodes. A solid line indicates a positive correlation, while a dotted line indicates a negative correlation. Nodes are colored based on log2-fold changes immediately post-flight compared to pre-flight time points. **c** The antioxidant defense subnetwork is enriched in peroxidases, antioxidant enzymes, and antioxidant molecules, indicating activation of extensive antioxidant response. **d** The immunosuppression and anti-inflammatory response subnetwork is enriched in anti-inflammatory molecules and protein markers of immune cells that lower post-flight, indicating a deregulated immune response. Source data are provided as a Source Data file.

We next investigated the common signature of oxidative stress and cellular detoxification found in EVP and plasma DAPs^35,36^ (Supplementary Fig. 3). Specifically, ROS scavenging is dependent on ROOH and H_2_O_2_ detoxification via superoxide dismutase (SOD1, SOD2), catalase (CAT), and peroxiredoxins^37,38^. Compared to ground controls, plasma from post-flight astronauts displayed an upregulation of antioxidant proteins and a distinct metabolic profile retained at both the immediate and long post-flight timepoints (Fig. 3a). Antioxidant proteins were also significantly enriched in EVPs in the intermediate post-flight timepoint, along with metabolic proteins involved in anabolic metabolism and cell growth, which may improve donor cells’ antioxidant capacity and cell bioenergetics^36,39–41^. Our findings indicate the body upregulates the production of anabolic metabolism and antioxidants in the plasma and EVPs, especially in the immediate-post-flight timepoint, likely to compensate for increased ROS (Fig. 3a). At the longterm post-flight timepoint, these protein levels are abolished in the EVPs, but not in the plasma, which maintains an upregulation of antioxidant proteins and an altered metabolic profile. These data indicate that intracellular ROS levels and metabolic profiles can remain differentially abundant following spaceflight for at least 80 days after landing.

### Integrating proteomics and metabolomics reveals a common signature of antioxidant defense and immune dysregulation

To investigate the molecular processes at the interface of altered proteins and metabolites, we undertook a correlation-based, integrated approach. Specifically, we correlated the proteins and metabolites across all timepoints, using the annotated DAPs and DAMs altered immediately post-flight (R + 1) compared to pre-flight (L-92, L-44, L-3). As a result, we identified 26 significant (False Discovery Ratio (FDR) < 5%) correlations between plasma proteins and plasma metabolites. In contrast, 1,416 correlations were significant between EVP proteins and plasma metabolites (Fig. 3b). The significantly correlated molecules were visualized as a network with proteins and metabolites as nodes, and the correlation between them as edges. Among these changes, two of the most commonly observed molecular changes post-flight were oxidative stress and immune dysregulation, as discussed below^1,22,42–47^.

First, we analyzed the interface between the ROS pathway and metabolites by correlating the proteins in the ROS pathway with metabolites. Exposure to radiation, microgravity, and hypoxia during spaceflight all induce the production of free radicals leading to oxidative stress, which can impact on cardiovascular, immune, neurological, and metabolic systems^1^. The ROS subnetwork from the i4 crew consisted of 26 nodes, including 6 proteins and 20 metabolites, with 38 correlation-based edges between the metabolites and proteins (Fig. 3c). All 6 proteins (100%) and 12 metabolites (60%) were lower post-flight, and 8 metabolites (40%) were higher post-flight. Within this subnetwork, three antioxidant enzymes from the peroxiredoxin family (PRDX1, PRDX2, PRDX6) which scavenge peroxides within cells^48^, were increased immediately post-flight. In addition, three other enzymes that degrade ROS, namely SOD1, CAT and glutamate-cysteine ligase (GCLC), were also increased post-flight. SOD1 catalyzes the conversion of superoxide into hydrogen peroxide, which CAT can then degrade^49^. GCLC is a rate-limiting enzyme for the de novo synthesis of glutathione, a widely studied antioxidant that maintains the cellular redox balance^50^. Moreover, antioxidants, including inosine and taurine, were significantly increased immediately post-flight (q-value < 0.05). In addition to its antioxidant capacities, inosine dampens cytokine production, normally ameliorating inflammation^51,52,6,53^. Taurine is also an antioxidant that protects immune cells during oxidative stress^54^, and its upregulation suggests that immediate post-flight antioxidant production compensates for spaceflight-induced oxidative stress.

We next analyzed the interface between the immune system and metabolism by correlating immune cell markers with specific metabolites. The immune subnetwork consisted of 56 nodes, including 10 proteins and 46 metabolites, with 95 correlation-based edges between the metabolites and proteins (Fig. 3d). All 10 proteins (100%) and 33 metabolites (71.7%) were lower immediately post-flight, and 13 metabolites (28.3%) were higher immediately post-flight. Within this subnetwork, all the protein markers of immune cells were decreased immediately post-flight. Moreover, our analysis indicated that anti-inflammatory and antioxidant molecules, namely inosine, purines, and taurine, were increased immediately post-flight. While taurine and inosine are antioxidants^51,54^, purines (e.g., adenosine) modulate the immune system by inhibiting the production of pro-inflammatory cytokines and free radicals^51^. In addition, 4-aminobutanoate (GABA), an immune-modulatory neurotransmitter, was also increased immediately post-flight, which can inhibit cytokine production^55,56^. This subnetwork may reflect widespread inflammation preceding the post-flight immunosuppression and anti-inflammatory responses, consistent with some of the NASA Twins Study results^22^ and studies of physiological stress, radiation, altered circadian rhythm^42,43^, and reactivation of latent herpes viruses^44,57^.

### Immune cells contribute to the observed secretome changes after spaceflight

**Fig. 4:**
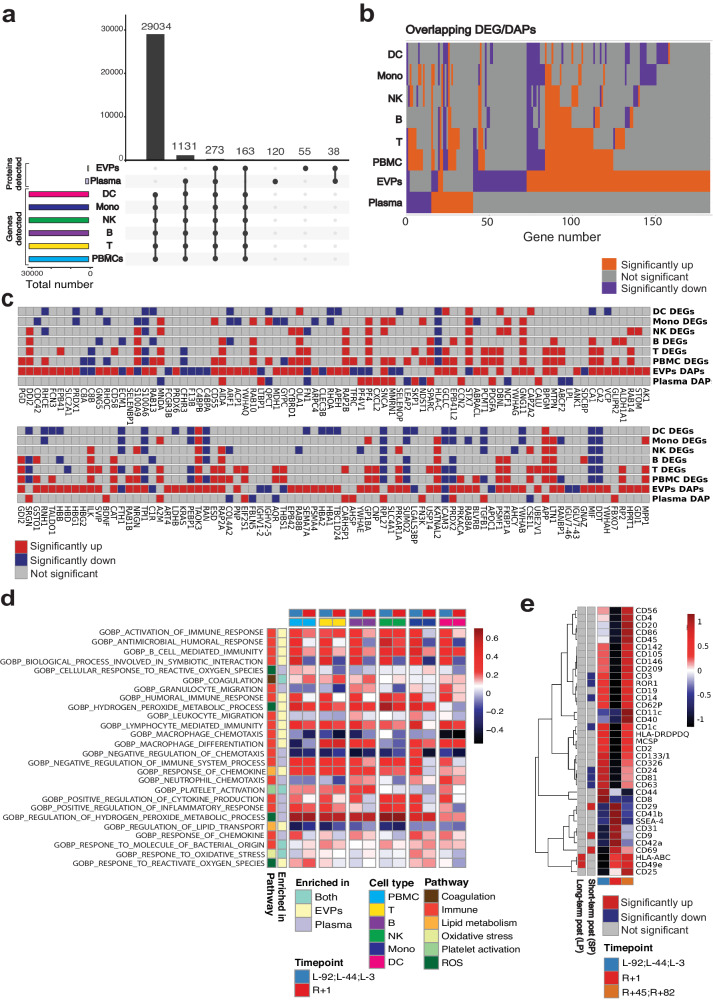
Immune cells contribute to the observed secretome changes after spaceflight. **a** Upset plot of identified genes in PBMC and identified proteins in EVPs and plasma. **b** Overlap of immune cell DEGs, plasma DAPs, and EVP daps. Up-regulated genes/proteins (Immune cells: *p*-value < 0.05, secretome: adjusted *p*-value < 0.05, fold change > 0) are depicted in orange. Down-regulated genes/proteins (Immune cells: *p*-value < 0.05, secretome: adjusted *p*-value < 0.05, fold change <0) are depicted in colored purple. Non-significant genes/proteins are depicted in colored gray. Wilcoxon rank sum tests were performed. **c** Expression of secretome DAPs in immune cells. **d** Fold change (R + 1/pre-flight) of the selected secretome-enriched pathways normalized score in immune cells. Among the secretome-enriched pathways, immune function, oxidative stress, antioxidant, lipid metabolism, coagulation, and platelet activation pathways were selected. **e** MACSPlex analysis of immune marker expression in EVPs. Source data are provided as a Source Data file.

To delineate the contribution of immune cells to the secretome, we compared our proteomic data to single nuclei gene expression of peripheral blood mononuclear cells (PBMCs) from the i4 crew using the 10X Genomics single-cell multi-ome kits for epigenetic and gene expression profiling (see *Methods*). Of the ~30,000 genes detected in PBMCs, 273 genes were detected in both plasma and EVPs (Fig. 4a). In addition, 1131 genes were uniquely reflected in the plasma proteome while 163 genes were uniquely found in the EVP proteome (Fig. 4a).

When comparing the differentially expressed genes (DEGs) of PBMCs with the plasma DAPs and EVP DAPs (Fig. 4a), we found that 12 (30%) plasma DAPs and 27 (17.8%) EVP DAPs were also differentially expressed in the PBMCs. Of those overlapping DAPs, 6/12 were differentially abundant in the same direction in both plasma and PBMCs. Additionally, 14/27 of the overlapping EVP DAPs were differentially abundant in the same direction in both EVPs and PBMCs. This likely indicates that circulating and EVP proteins reflect gene expression changes in immune cells, while validating that secreted proteins can also originate from non-immune cells and distal organs.

To further examine the connection between the EVP proteome and the PBMCs transcriptional states, we examined the overlap between EVP DAPs and PBMC DEGs. The crew EVP profiles showed a higher overlap with the DEGs among lymphoid cells (T cell, B cell, Natural Killer (NK) cell) than with myeloid cells (Fig. 4b), and the same trend was observed for plasma DAPs (Fig. 4b), indicating that lymphoid cells contributed more than myeloid cells to the observed changes in the secretome. Among the genes differentially abundant in EVPs and PBMCs, we noted several immune genes such as Integrin linked kinase (ILK), which was upregulated in EVPs and PBMCs, while carbonic anhydrase 8 (C8A) and complement C8 beta chain (C8B) were downregulated in EVPs and PBMCs (Fig. 4c).

Among the antioxidant and oxidative stress-related proteins, PRDX2 was again upregulated in both plasma and EVPs, but was downregulated in DC cells, Monocytes, T cells and B cells, indicating that the upregulation of PRDX2 and EVPs seen in plasma does not originate from immune cells (Fig. 4c). To disentangle the relationship between immune cells and the pathways enriched based on the secretome DAPs, we calculated the fold changes of normalized enrichment score (NES) of immune cells at R + 1 versus pre-flight for the significantly enriched secretome DAPs pathways related to coagulation, immune function, lipid metabolism, oxidative stress, platelet activation, and reactive oxygen stress (Fig. 4d and Supplementary Fig. 3). We found an enrichment in oxidative stress and ROS pathways in T cells, NK cells, monocytes, and DC at R + 1 compared to pre-flight timepoints.

To gain insight into potential cellular sources of plasma EVPs, we used the MACSPlex Exosome profiler, which estimates the abundance of EVs expressing one of 37 markers specific for various immune cell types. We found significant increases in the pan-EVP marker CD9^58,59^, integrin beta-1 (ITGB1, also CD29)^60^, and B/T activation marker (CD69)^61^ and significant decreases in Alveolar Type I/Brain (Receptor tyrosine kinase like orphan receptor, ROR1)^62–64^, melanocytes (Melanoma chondroitin sulfate proteoglycan, MCSP)^65,66^, T cells (CD3)^67^, additional pan-EVP markers (CD63^68^, CD81), B cell (CD24)^69^, and DC (CD1C)^70^ markers (Fig. 4e). The increase in CD9 at R + 1 correlates with an overall increase in EVP production post-flight^15^, while the decrease in CD63 and CD81 at R + 1 is consistent with the increase in CD9+ EVPs produced by platelets involved in coagulation. Of note, CD69 (a T and B cell activation marker) was increased at R + 1, consistent with inflammation revealed by the other omics analyses. The increase in ITGB1 (CD29), which complexes with integrin subunit alpha 5 (ITGA5, also CD49e)^71^, a heterodimer expressed on activated lymphocytes, endothelial cells (ECs), osteoblasts and which binds fibronectin and L1 cell adhesion molecule (L1CAM, a central nervous system axonal protein), may be consistent with vascular permeability, and systemic inflammation. Several DC markers, CD24, CD1c, and CD209, were also decreased at R + 1, consistent with suppressed DC function.

We hypothesized that the significantly changed immune markers in EVPs would overlap with the i4 immune cell DEGs (Supplementary Fig. 4). Indeed, we found that while DEGs in PBMCs were driven more by T and B cells, all cell types showed changes in vesicle regulation (Supplementary Fig. 5). CD3 delta, a pan-T cell marker, was downregulated in PBMCs, T cells, and their EVPs, whereas a different T cell marker (CD69) was downregulated in cells, but enriched in EVPs. This could indicate that CD69 is selectively shuttled into EVPs, or selectively enriched in EVPs derived from tissue-resident memory T cells. Moreover, CD63 was consistently downregulated in innate immune cells (DC, NK, macrophages) and EVPs immediately post-flight, indicating that cellular reduction was responsible for decreased EVP CD63 levels.

### Red blood cells do not contribute to secretome changes after spaceflight

To delineate the contribution of blood cells to the secretome, we compared the gene expression profiles of whole blood direct RNA-seq data (Oxford Nanopore) from the i4 crew and identified 61 overlapping DEGs. We then compared the gene list with the plasma and EVP DAPs (Supplementary Fig. 6a, b). Interestingly, protein abundances in EVPs and gene expression in whole blood were inversely correlated (increased in EVPs and decreased in whole blood). The expression of these genes in PBMCs, however, was mostly aligned with the protein abundance, with at least one cell type showing a significant increase in PBMCs for SNCA. Of note, SNCA overlapped in whole blood DEGs, plasma DAPs, and EVP DAPs.

Overall, 8 genes were shared between whole blood DEGs and EVP DAPs and one gene was shared between blood DEGs and plasma DAPs. To determine the contribution of whole blood and PBMC to the secretome, we then examined expression of these overlapping DAPs (AHSP, AK1, ANK1, BLVRB, EPB42, HBD, ENBP1, SNCA) before and after spaceflight (Supplementary Fig. 6c), and analyzed this list for enriched gene functions. Significant enrichment (q < 0.01) was observed in heme metabolism, anemia, hematologic disease, brain function (terminal button, axon part)-related pathways (Supplementary Fig. 6d). The gene SLC4A1 was present in nearly all overrepresented groups (excluding the cell cortex and axon part), indicating a brain-related phenotype that warranted further investigation.

### Brain-related signatures increased in the secretome after spaceflight

Spaceflight exposes the human brain to several stressors, which have the potential to cause short-term and long-term neurological effects, including SANS, body fluid shift, neuroinflammation, and neurodegeneration^12,72–74^. In addition, an increasing body of evidence suggests that the spaceflight environment could induce blood-brain barrier (BBB) disruption^72,75–78^. For example, EVP proteomic profiles of NASA Twins study obtained three years post-return from a year-long flight revealed brain-associated proteins in the plasma of the astronaut twin, but not the ground control twin^15^.

**Fig. 5:**
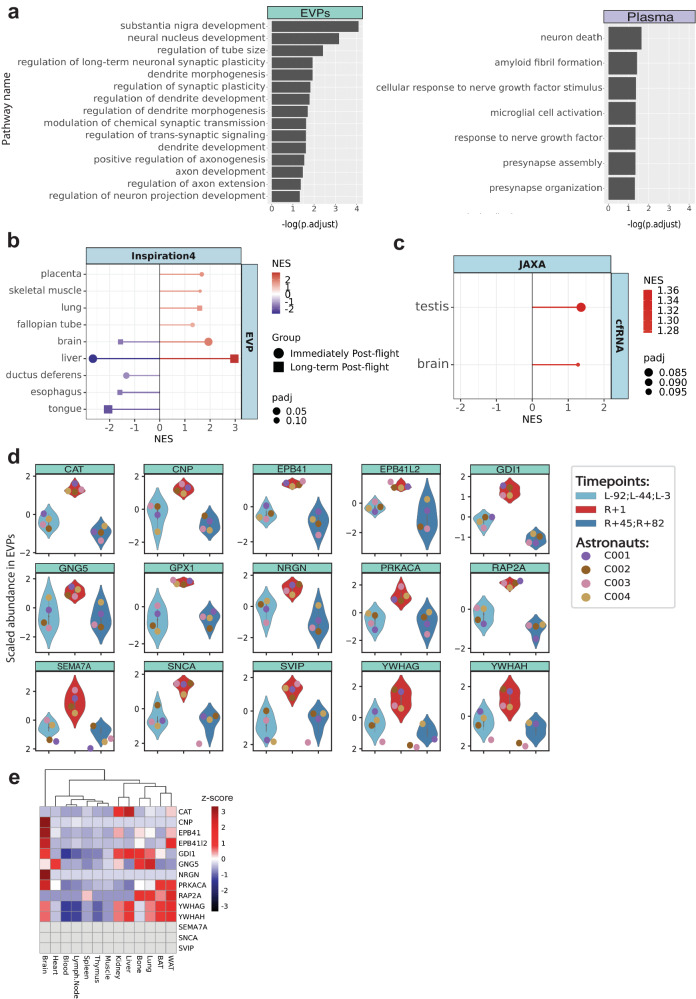
Brain-related proteins are enriched in the secretome after spaceflight. **a** Overrepresentation analysis of significantly enriched pathways (adjusted *p*-value < 0.05) related to brain function and injury of EVPs and plasma DAPs at R + 1 (adjusted p-value < 0.05, Left: EVP, Right: plasma). **b** Gene set enrichment analysis (GSEA) of EVP DAPs immediately post-flight and long-term post-flight based on the tissue-enriched database derived from the Human Protein Atlas database. GSEA was performed with fgsea::fgsea() using minSize=5 and maxSize = 500 as parameters. Significant results (adjusted *p*-value < 0.1) are shown. **c** Gene set enrichment analysis of cfRNA measured immediately post-flight JAXA CFE mission based on the tissue-enriched database derived from the Human Protein Atlas database. GSEA was performed with fgsea::fgsea() using minSize = 5 and maxSize = 500 as parameters. Significant results (adjusted *p*-value < 0.1) are shown. **d** Abundance of brain-enriched proteins in EVPs. Data is from n = 4 astronauts, representing one technical replicate per astronaut and timepoint averaged at the indicated condition (preflight, and long-term postflight). Boxes show the quartiles of the dataset while the whiskers extend to show the rest of the distribution except for “outliers”. Source data are provided as a Source Data file. **e** Abundance of brain-enriched proteins in EVPs isolated from naive, ground control mice.

Since EVPs are known to be released from distal organs such as the brain, and could be detected in plasma, we examined post-spaceflight EVPs for any enrichment of brain-specific or brain-associated proteins. Indeed, both EVPs and plasma DAPs were enriched for brain function and brain injury-related pathways (Fig. 5a), including neurodegeneration pathways, neuron death, and amyloid fibril formation. Moreover, Gene Set Enrichment Analysis (GSEA) analysis revealed that brain-associated proteins were increased in plasma at R + 1 (Fig. 5b**)**, which matches orthogonal data from a JAXA cfRNA-seq study that also revealed an increase in brain-enriched proteins immediately post-flight (R + 3) (Fig. 5c) (study OSD-530 of 6 Japanese astronauts on the International Space Station, ISS). Of note, the spike in brain signatures for plasma, cfRNA, and exosome proteins was most pronounced in the days after landing back on Earth (R + 1 and R + 3) (Fig. 5d).

To examine possible sources of spaceflight-associated brain signatures in plasma, two hypotheses were examined: (1) proteins are purposely packaged and shuttled from the brain into EVPs and released or (2) BBB integrity is disrupted, indicating “leakiness” of brain proteins. To address the first hypothesis, we examined EVP protein cargo data in 13 mouse tissues from a public EVP atlas^25^ (blood, thymus, lymph node (LN), brown adipose tissue (BAT), bone, brain, heart, kidney, liver, lung, spleen, white adipose tissue, and muscle). Of the 16 brain-annotated proteins, 3 were brain-exclusive (CNP, EP41, NRGN), 2 were highly enriched in the brain (Epb4L1 and PRKACA), and five others were highest in the liver. However, SNCA, semaphorin 7 A (SEMA7A), and small VCP-interacting protein (SVIP) were not detected in mouse brain EVPs, nor in other tissues (Fig. 5e), indicating that packaging of these proteins into EVPs is unlikely, given their absence in the murine brain tissue.

**Fig. 6:**
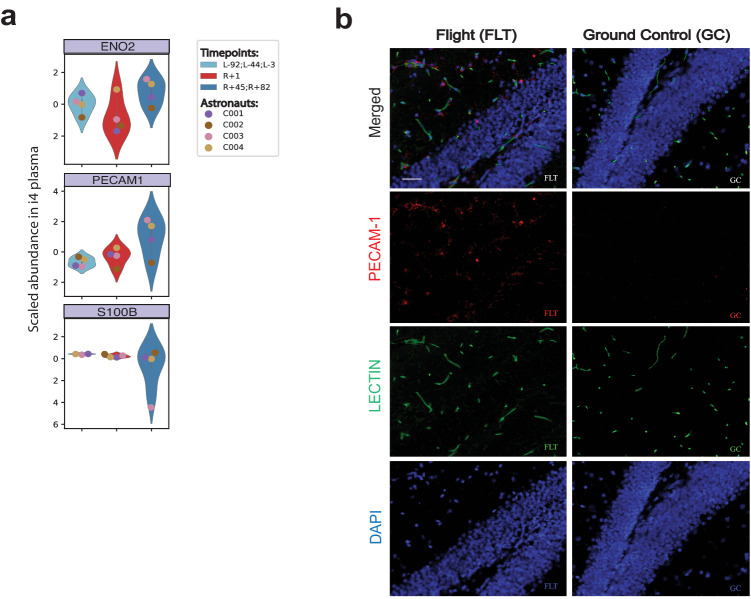
Blood Brain Barrier integrity markers in spaceflight. **a** Abundance of blood-brain barrier (BBB) integrity peptides in plasma of i4 astronauts shown as violin plots. Data is from *n* = 4 astronauts. Each blood proteomic measurement was performed in two technical replicates per astronaut and timepoint. Displayed data represents the average of the technical replicates which were further averaged at the indicated condition (preflight, and long-term postflight). Boxes show the quartiles of the dataset while the whiskers extend to show the rest of the distribution except for “outliers”. **b** Representative images of hippocampal PECAM-1 in the flight (FLT) and ground control (GC) mice (*n* = 5). PECAM-1 positive cells were identified based on red fluorescence, while endothelium was stained with lectin (green). The nuclei were counterstained with DAPI (blue). In the control hippocampal region, few positive cells were found. In the hippocampal region of FLT mice, enhanced PECAM expression could be detected. There was a significant difference between FLT and GC groups with *p* < 0.05 (*n* = 5 replicates, two-sided Student’s paired t-Test, *p* = 0.023).Source data are provided as a Source Data file. Scale bar = 50 mm.

To test the alternative hypothesis of BBB disruption, we examined the expression of biomarkers previously associated with BBB integrity, specifically S100 calcium binding protein B (S100B), Enolase 2 (ENO2) and Platelet Endothelial Cell Adhesion Molecule (PECAM-1)^79,80^ (Fig. 6a, b). While the proteins ENO2 and S100B showed no significant difference for pre/post-flight, PECAM-1 showed an increase in the plasma protein abundance at R + 1 in C001, C003, and C004, and a postflight increase as well when measured at R + 45 and R + 82 (Wilcoxson rank sum, *p* = 0.07)(Fig. 6a). To further examine the in vivo changes in PECAM-1, we used brain tissue from rodents flown on the RR-18 mission, which spent 35 days on the ISS. After spaceflight, the RR-18 rodents were returned to Earth, wherein the flight samples (FLT) were dissected and fixed onto slides for straining at the same time as the ground controls (GC). Interestingly, significantly increased PECAM-1 immunoreactivity (*n* = 5 replicates, two-sided Student’s paired t-Test, *p* = 0.023) was detected in the FLT samples relative to the GC group (Fig. 6b), implicating PECAM-1 as a possible spaceflight-related marker for BBB integrity.

## Discussion

Secretome profiling of the i4 crew after a 3-day spaceflight revealed significant changes in oxidative stress, brain homeostasis, and coagulation markers. These changes largely recovered post-spaceflight, although some proteins (particularly in plasma) still remained differentially abundant six months later. Although this study focuses on a short-term mission, our findings recapitulate several of the responses observed in the NASA Twins study including anemia, coagulation, and oxidative stress, as well as spaceflight-associated brain homeostasis alterations^15,22^, which indicate recurrent biological perturbations relevant for future crews and missions. Multiple studies have reported a higher risk of developing internal jugular vein thrombosis in the ISS crews than the general population, a condition that may lead to pulmonary embolism and long-term morbidity^13,14,81^.

Fortunately, the overall risk of spaceflight-induced clotting returns to baseline upon landing, as indicated by normal levels of pro-thrombotic factors PF4 and PF4V1 in EVPs and plasma collected 82 days post-flight^13,14^. Most metabolic alterations induced by spaceflight were also temporary, with metabolic profiles rapidly returning to their pre-flight states upon return to Earth. Nonetheless, the 100 specific altered metabolites from spaceflight showed enrichment for oxidative stress, hemolysis, lipid peroxidation, and immune suppression pathways. These pathways included metabolites that have appeared in a previous study (the Soyuz-36-Salyut-6-Soyuz-35 mission), such as 1-methylinosine^82^. A 45-day head-down tilt study showed dynamic changes in taurine, glycine, betaine, creatine, and glutamine^83^, which also mirrored results in mice^84^, and taurine and inosine demonstrated correlations with several antioxidant enzymes.

Indeed, in our proteomics data, we observed upregulation of several antioxidant proteins such as PRDX2, SOD2, CAT, and GPX1. PRDX2 increased in all crew members immediately post-flight (R + 1), while PRDX6 and SOD2 were decreased, and 2 proteoforms of catalase were increased in one crewmember after landing. A spaceflight mouse study also observed PRDX6 and catalase gene upregulation in skin samples^85^, while a tail suspension rat study observed upregulation of PRDX6 in the hippocampus^86^. Additional rodent studies have also shown changes in oxidative stress proteins (SOD1, SOD2, Xanthine oxidase, etc.) resulting from spaceflight factor exposure, both post-exposure and longterm^86,87^. Thus, we propose that the oxidative stress induced by spaceflight triggers the production of both proteins and metabolites with antioxidant functions. Signs of hemolysis were also evident, underscored by an increase in circulating protoporphyrin, a known marker of this process. The observed decrease in S1P plasma abundance could also signal a reduction in erythrocytes, cells responsible for maintaining S1P concentrations in the blood. Our data further indicated lipid peroxidation, a biochemical process known to disrupt cellular membrane stability and decrease cell viability. Complementing these findings, we found an enrichment in anemia-related genes in our whole blood transcriptomics data and signs of coagulation dysregulation, often associated with anemia, in both EVPs and plasma proteomics. Indeed, spaceflight anemia has been previously observed and reported, consistent with our observations^87^. Lastly, evidence of immune suppression was apparent. Aside from their roles as antioxidants, inosine and taurine possess immunoprotective properties. Along with the immunomodulatory metabolite GABA, these metabolites correlated with immune cell markers. Given that the abundance of these immune cell markers was low while immune modulatory metabolites were in high abundance, we hypothesize an activation of immunosuppression and anti-inflammatory responses after inflammation during the flight^88^.

Our analysis of the i4 crew revealed a notable increase in several brain-associated proteins within both plasma and EVPs following spaceflight. Notably, SNCA was significantly increased in plasma, EVPs, and several immune cell types immediately after spaceflight, suggesting it represents a common protein marker of response to spaceflight. SNCA is associated with Parkinson’s disease, and elevated levels have been linked to brain dysfunction and neurodegenerative disorders like Parkinson’s disease^89^. Therefore, understanding these protein changes can guide the development of targeted therapeutic strategies for managing neurodegenerative diseases in space and on Earth. However, further investigations are necessary to better understand the precise mechanisms and implications of changes in brain-associated proteins as well as means to modify their levels safely.

Although these brain-associated proteins suggest some dysregulation of homeostasis in the brain, none of these proteins are unique to the brain, and thus it is possible that they may originate from other organs. Correlations between expression of these proteins in the brain and in the blood in response to spaceflight is required to confirm whether increased levels reflect dysregulation in the brain. However, a similar increase in brain-enriched transcripts/proteins was observed in JAXA Cell free epigenome (CFE) cfRNA (more than 120 days on the ISS), and the NASA Twins study EVP proteome, (1-year on the ISS) in-flight and immediately post-flight. We note that the amplitude and durability of changes in brain homeostasis and potential BBB disruption/neuroinflammation may be dictated by the length of time spent in space or distance from Earth. Previous reports of space flown mice on brain state have shown decreased brain derived neurotrophic factor, induced neuron atrophy in the cerebral cortex, and overall oxidative stress, regulated partially through c-Jun/c-Fos^90^. Moreover, a recent MRI-based study of astronaut brains found that spaceflight induced ventricular expansion in the brain^91^. Finally, increased hippocampal apoptosis and aquaporin-4 expression have been observed in other studies^75^, along with significant increase in the expression of PECAM-1 and decreases in the BBB-related tight junction protein, Zonula occludens-1 (ZO-1). Given the murine and human data observed to date, as well as data from this paper, PECAM-1 is a strong candidate as a BBB biomarker that could be measured in upcoming missions.

Overall, secretome profiling provides a minimally invasive, yet comprehensive approach to monitor crew health and physiology. Based on this work and follow-up studies, we propose to develop a liquid biopsy and biomarker panel to monitor space-associated health risk and link these to long-term work on countermeasures. Eventually such tools can generate reliable markers in response to spaceflight for more diverse populations (age, sex, health, background). Due to the limitation of the Dragon capsule equipment, we were unable to obtain in-flight secretome data for the i4 study, which would also be critical to study in the future. Additionally, creating more profiles from other crews and controls will help build a larger, more informative cohort, and further buttress and give context to the results seen in these data. Nonetheless, as human spaceflight becomes more prevalent, the necessity for deep secretome profiling can help establish baseline profiles for safer human space travel increases, and these tools can be part of the armamentarium of biomedical tools to help keep crews safe for upcoming, exploration-class missions, as well as continued health on Earth.

## Methods

### Blood pre-processing and EVP (extracellular vesicle and particle) isolation

Detailed descriptions of the methods pertaining to all proteomics/metabolomics/transcriptomics assays mentioned in this paper can be found in the “protocol” section of the GeneLab OSDR links found below^92^. Briefly, blood was collected in K2 EDTA tube(s) and shipped overnight on ice. Plasma was isolated by differential centrifugation at 500 x g for 10 minutes and 3000 x g for 20 min as previously described in refs. ^24,93^. The supernatant was collected and aliquoted for long-term storage at −80 °C and for EVP isolation. Plasma EVPs were isolated by sequential ultracentrifugation, as previously described in refs. ^25^. EVP protein concentration was measured by bicinchoninic acid (BCA) protein assay (Pierce, Thermo Fisher Scientific).

Additional sample collection methods and data generation have been also detailed in the protocols paper^24^.

### Plasma proteomic profiling

Detailed methods for proteomic, metabolic and transcriptomic profiling are described in Overbey et al. 2024. Briefly, plasma was isolated from cell preparation tubes (CPTs) and processed with Seer’s Proteograph Analysis Suite^23^. Seer’s standard 5-nanoparticle (5-NP) panel was used to enrich low abundance proteins followed by LC-MS/MS proteomics analysis in data-independent acquisition (DIA) mode. The list of differentially abundant proteins from the I4-FP1 and I4-FP4 plasma proteomics dataset in Overbey et al. 2024 was filtered for differentially expressed genes that had an adjusted *p*-value < 0.05 and |logFC | > 1^92^.

### Extracellular vesicles and particle (EVP) proteomic profiling

EVP proteomic profiling was described in Overbey et al. 2024^92^. Briefly, plasma samples were centrifuged at 12,000 x g for 20 minutes and then EVPs were collected by ultracentrifugation at 100,000 x g for 70 min. EVPs were then washed in PBS and again collected by ultracentrifugation at 100,000 x g for 70 min. The final EVP pellet was resuspended in PBS. Two micrograms of enriched EVPs were digested and analyzed with LC-MS/MS in data-dependent acquisition (DDA) mode. The list of differentially abundant proteins from the I4-FP1 and I4-FP4 plasma proteomics dataset in Overbey et al. was filtered for differentially expressed genes that had an adjusted *p*-value < 0.05 and |logFC | > 1^92^.

### Plasma metabolite profiling

Plasma metabolite profiling was described in Overbey et al. 2024^93^. Briefly, plasma metabolites were isolated using a combination of aqueous normal phase (ANP) and reverse phase (RP) chromatographic separations and analyzed by positive- and negative-ion MS. Metabolites were annotated using an in-house metabolite database comprising 865 metabolites for ANP chromatography and a 270 lipid metabolite RP database, curated from the Agilent plasma lipidomic database. All metabolites identified are level 1. Namely, to be identified, the feature’s mass, chromatographic retention time (RT) and MS/MS fragmentation pattern had to match to measurements acquired from the analysis of a pure chemical reference standard in our lab’s in-house metabolite database. The list of differentially abundant metabolites from the I4-FP1 and I4-FP4 plasma proteomics dataset in Overbey et al. 2024 was filtered for differentially expressed genes that had an adjusted p-value < 0.05 and |logFC | > 1^93^.

### Western blot validation of EVP markers

We validated DEPs in human plasma-derived EVPs by immunoblotting. For this, 5 µg of EVP protein were subjected to denaturing electrophoresis (SDS-PAGE), transferred onto a PVDF membrane and incubated overnight at 4 °C with primary antibodies against human Galectin 3 Binding Protein (LGALS3BP) (mouse monoclonal IgG, 1:1000 dilution, sc-374541, Santa Cruz Biotechnology) and Ficolin-3 (FCN3) (rabbit polyclonal IgG, 1:500 dilution, 11867-AP, Proteintech). Secondary antibodies used were horseradish peroxidase (HRP)-labeled IgG goat anti-rabbit or goat anti-mouse (1 h incubation, 1:5000 dilution, Jackson Laboratory). All antibodies were diluted in 5% BSA in Tris-buffered saline with 0.1% Tween-20 (Thermo Fisher Scientific). The immunocomplexes were developed using the SuperSignal™ Western Blot Enhancer (Pierce, Thermo Fisher Scientific) and the membranes were imaged using a ChemiDoc Imaging System (Bio-Rad).

### ELISA validation of plasma markers

PF4 concentration in EVP-rich plasma (post-3000 x *g* spin) and EVP-depleted plasma (CPT tubes, see proteomic mass spectrometry analysis) was assayed with a PF4 ELISA kit (R&D, cat #DPF40) according to the manufacturer’s instructions.

### Thiobarbituric acid reactive substances (TBARS) assay

To assess the lipid peroxidation in plasma, we measured the levels of TBARS as by-products of lipid peroxidation using the Lipid Peroxidation (MDA) Assay Kit (Abcam, ab118970), according to the manufacturer’s instructions. For this assay, 10 µl of plasma were used for generating malondialdehyde (MDA) adducts with thiobarbituric acid (TBA) which were further quantified fluorometrically (Ex/Em = 532/553) against a standard curve ranging between 0-0.5 nmol TBARS/well. Data were analyzed according to the manufacturer instructions taking into consideration the plasma volume used for the assay and results were reported as µM TBARS. Repeated measures one-way ANOVA was performed to assess significance.

### Multiplex bead-based characterization/profiling of plasma EVPs by flow cytometry

The relative abundance of specific, immune-related plasma EVP proteins, plasma EVPs enriched by sequential ultracentrifugation (1 µg of EVP protein) were characterized using the MACSPlex Exosome Kit (Miltenyi Biotec, 130-108-813) according to the manufacturer instructions for overnight protocol for 1.5 ml reagent tubes. Data was acquired on a Cytek Aurora-5 (Cytek Biosciences, USA) instrument and analyzed using the FlowJo™ v10.8 Software (BD Biosciences). The relative abundance (represented as MFI) for each EVP epitope was normalized against the mIgG and the average MFI of EV markers (CD9, CD63, and CD81), and the results were displayed as a heatmap. Welch’s two sample t-test were used to calculate P-value. Heatmaps were generated using the R (v4.1.2) package pheatmap (v1.0).

### Single-nuclei gene expression analysis of peripheral blood mononuclear cells

Detailed methods for sample collection and data processing are described in refs. ^93,94^. Blood samples were collected before (Pre-launch: L-92, L-44, and L-3) and after (Return; R + 1, R + 45, and R + 82) the spaceflight. Chromium Next GEM Single Cell 5’ v2, 10x Genomics was used to generate single cell data from isolated PBMCs. We followed the analysis pipeline as previously reported^95^. Subpopulations were annotated based on Azimuth human PBMC reference. Gene expression values were used to generate heatmaps. Selected pathways were used for the ssGSEA analysis.

### Direct RNA-sequencing on Oxford Nanopore Technologies PromethION

The list of differentially expressed genes from the I4-FP1 direct RNA-sequencing dataset in Overbey et al. 2023 (In review at Nature) was filtered for differentially expressed genes that had an adjusted p-value < 0.05 and |logFC | > 0.5. Briefly, these differentially expressed genes were identified using Oxford Nanopore Technologies package pipeline-transcriptome-de^96^. Intervene was used to identify overlaps between differentially expressed genes and differentially abundant proteins in EVP and plasma datasets and create the Venn diagram^97^. PBMC gene expression data was obtained from the i4 PBMC single cell data. Direct RNA-seq gene expression, proteomic abundances, and PBMC gene expression values were normalized by pre-flight values. Overrepresentation analysis was performed with WebGestalt^98^.

### Integration of metabolomics and proteomics

For the correlation-based network analysis, we used protein/metabolite pairs that showed a significant Spearman correlation (FDR < 5%) across all time points. In this network, proteins are depicted by ellipses, while metabolites are represented by square nodes. Edges between them signify significant correlation: a solid edge indicates a positive correlation, and a dotted edge signifies a negative one. The analysis considered differentially abundant proteins and metabolites that changed immediately post-flight (R + 1) compared to pre-flight (L-92, L-44, L-3) to compute these correlations. Furthermore, the color of each node reflects the log fold change of that node post-flight (R + 1).

### Spaceflight and Mouse Groups

Ten-week-old C57BL/6 male mice were launched in December 2021 to the international space station (ISS) on the rodent research-18 (RR-18) mission for 35 days. All mice were maintained at an ambient temperature of 26–28 °C and humidity of 30–70% with a 12 h light/dark cycle during the flight. This hardware has a housing density that is within the guidelines recommended by the National Institutes of Health. All mice were provided NASA Nutrient-upgraded Rodent Food Bar (NuRFB) and autoclaved deionized water ad libitum. Ground control (GC) mice were maintained on Earth in the same flight hardware cages. Upon live return, mice were exsanguinated by closed-cardiac blood collection under deep Ketamine/Xylazine (150/45 mg/kg) anesthesia, followed by cervical dislocation as a secondary euthanasia method to ensure death. Their brains were removed and prepared for analysis. The left hemi-brains were fixed in 4% paraformaldehyde in phosphate-buffered saline (PBS) for 24 h, and then rinsed with PBS for immunohistochemistry (IHC) assays. The right hemi-brains were flash-frozen and stored at −80 °C for further analysis. GC mice were euthanized three days later. Animal experiments were approved by the National Aeronautics and Space Administration (NASA) Animal Care and Use Committee (IACUC) on October 14, 2021 (Protocol Number: RR-18), Roskamp Institute IACUC on October 7, 2021 (Protocol Number RR-18).

### Immunostaining assays for PECAM-1

Brain sections were immunofluorescence stained against PECAM-1, a biomarker related to BBB. Six µm sections were deparaffinized, rehydrated and washed in PBS for 20 min. Vascular network was labeled with DyLight® 488 Lycopersicon Esculentum (Tomato) Lectin (1:100, Vector Laboratories) for 30 min at room temperature followed by 10 min wash in PBS. Sections were then incubated overnight at 4 °C with primary rabbit antibodies PECAM-1 (1:100, NB100-2284, Novus Biologicals, Centennial, CO). After 3 washes in PBS, sections were incubated for 1.5 h with secondary antibody goat anti-rabbit IgG Alexa Fluor® 568 (1:1000 in antibody dilution buffer; Life Technologies). The cell nuclei were counterstained with DAPI solution (Life Technologies) and coverslipped with Vectashield® HardSet mounting medium (Vector Laboratories). Six to 10 field images were captured with a BZ-X700 inverted fluorescence microscope (Keyence Corp.) at 20X magnification spanning the entire brain sections.

### Mouse Tissue EVP isolation and proteomic analysis

Tissues were isolated from 6 to 8 week-old naive female C57BL/6 mice and processed as previously described in ref. ^25^. EVPs were isolated and unbiased proteomic profiling of EVP cargo was performed as described above. Mouse studies were performed in accordance with institutional, IACUC and AAALAS guidelines, and according to Weill Cornell Medicine animal protocol #0709-666 A.

### Tissue of origin deconvolution analysis

To perform tissue of origin deconvolution, a list of proteins “enriched” or “specific” to 36 different tissues compiled from the Human Protein Atlas (https://www.proteinatlas.org/) was used as pathway input to fgsea (version 1.22) with a minimum size=5 and maximum size=500. The list of differentially abundant features was ranked based on the t-statistic and used as gene list input. We have added the script used for this analysis to the github repository.

The file corresponding to the 36 tissues was generated from the data in the link below:

https://www.proteinatlas.org/humanproteome/tissue/tissue+specific which include 36 tissues.

### IRB statement human subjects research

All subjects were consented at an informed consent briefing (ICB) at SpaceX (Hawthorne, CA), and samples were collected and processed under the approval of the Institutional Review Board (IRB) at Weill Cornell Medicine, under Protocol 21-05023569. All crew members have consented for data and sample sharing. Tissue samples were provided by SpaceX Inspiration4 crew members after consent for research use of the biopsies, swabs, and biological materials. The procedure followed guidelines set by the Health Insurance Portability and Accountability Act (HIPAA) and operated under Institutional Review Board (IRB) approved protocols. Experiments were conducted in accordance with local regulations and with the approval of the IRB at Weill Cornell Medicine (IRB #21-05023569).

### Reporting summary

Further information on research design is available in the Nature Portfolio Reporting Summary linked to this article.

### Supplementary information

Supplementary Information

Peer Review File

Reporting Summary

### Source data

Source Data

## Data Availability

Source data of the figures are provided with this paper. All datasets in this paper have been deposited in the NASA Open Science Data Repositories (OSDR; osdr.nasa.gov; comprised of GeneLab^92^ and the Ames Life Sciences Data Archive [ALSDA]^1,99^). Identifiers for publicly downloadable datasets in the OSDR are documented below. Also, data can be visualized online through the SOMA Data Explorer (link below) with the latest reference^100^. Any additional information required to reanalyze the data reported in this work is available from the Lead Contact upon request. For the blood plasma, the following assays are found under OSDR identifier OSD-571: Proteomics (Seer Proteograph), proteomics of blood EVPs, plasma metabolomics data, cell-free RNA. Direct RNA-seq from blood plasma can be found under OSDR identifier: OSD-569 (Supplementary Table 1) All raw data, processed data and detailed methods are in the GeneLab OSDR links here: https://osdr.nasa.gov/bio/repo/data/studies/OSD-569https://osdr.nasa.gov/bio/repo/data/studies/OSD-571 Interactive data browser for the proteomics, metabolomics, and other omics data are available on the SOMA data portal: https://soma.weill.cornell.edu/apps/SOMA_Browser/ Source data are provided with this paper.

## Code availability

All code used to generate the analyses in this manuscript are available on GitHub at https://github.com/eliah-o/inspiration4-omics.

## References

[1] Afshinnekoo E (2020). Fundamental Biological Features of Spaceflight: Advancing the Field to Enable Deep-Space Exploration. Cell.

[2] Kliuchnikova AA (2023). Blood Plasma Proteome: A Meta-Analysis of the Results of Protein Quantification in Human Blood by Targeted Mass Spectrometry. Int. J. Mol. Sci..

[3] Brzhozovskiy AG (2019). The Effects of Spaceflight Factors on the Human Plasma Proteome, Including Both Real Space Missions and Ground-Based Experiments. Int. J. Mol. Sci..

[4] Larina IM (2017). Protein expression changes caused by spaceflight as measured for 18 Russian cosmonauts. Sci. Rep..

[5] Martin D, Makedonas G, Crucian B, Peanlikhit T, Rithidech K (2022). The use of the multidimensional protein identification technology (MudPIT) to analyze plasma proteome of astronauts collected before, during, and after spaceflights. Acta. Astronaut.

[6] Kashirina DN (2020). Semiquantitative Proteomic Research of Protein Plasma Profile of Volunteers in 21-Day Head-Down Bed Rest. Front. Physiol..

[7] da Silveira WA (2020). Comprehensive Multi-omics Analysis Reveals Mitochondrial Stress as a Central Biological Hub for Spaceflight Impact. Cell.

[8] Hoshino A (2020). Extracellular vesicle and particle biomarkers define multiple human cancers. Cell.

[9] Liang Y, Lehrich BM, Zheng S, Lu M (2021). Emerging methods in biomarker identification for extracellular vesicle-based liquid biopsy. J. Extracell. Vesicles.

[10] Vaughn MN, Winston CN, Levin N, Rissman RA, Risbrough VB (2021). Developing Biomarkers of Mild Traumatic Brain Injury: Promise and Progress of CNS-Derived Exosomes. Front. Neurol..

[11] Xu K (2020). Extracellular vesicles as potential biomarkers and therapeutic approaches in autoimmune diseases. J. Transl. Med..

[12] Chakrabortty SK (2022). Exosome based analysis for Space Associated Neuro-Ocular Syndrome and health risks in space exploration. NPJ Microgravity.

[13] Limper U (2021). The thrombotic risk of spaceflight: has a serious problem been overlooked for more than half of a century?. Eur. Heart J..

[14] Auñón-Chancellor SM, Pattarini JM, Moll S, Sargsyan A (2020). Venous Thrombosis during Spaceflight. N. Engl. J. Med..

[15] Bezdan D (2020). Cell-free DNA (cfDNA) and Exosome Profiling from a Year-Long Human Spaceflight Reveals Circulating Biomarkers. iScience.

[16] Morukov BV, Nichiporuk IA, Tret’yakov VS, Larina IM (2005). Biochemical Markers of Bone Tissue Metabolism in Cosmonauts after a Prolonged Spaceflight. Hum. Physiol..

[17] Channon MB (2015). Using natural, stable calcium isotopes of human blood to detect and monitor changes in bone mineral balance. Bone.

[18] Smith JK (2018). IL-6 and the dysregulation of immune, bone, muscle, and metabolic homeostasis during spaceflight. NPJ Microgravity.

[19] Smith SM, Heer M (2002). Calcium and bone metabolism during space flight. Nutrition.

[20] Ferrando AA, Paddon-Jones D, Wolfe RR (2002). Alterations in protein metabolism during space flight and inactivity. Nutrition.

[21] Smith SM (2002). Red blood cell and iron metabolism during space flight. Nutrition.

[22] Garrett-Bakelman FE (2019). The NASA Twins Study: A multidimensional analysis of a year-long human spaceflight. Science.

[23] Blume JE (2020). Rapid, deep and precise profiling of the plasma proteome with multi-nanoparticle protein corona. Nat. Commun..

[24] Overbey EG (2024). Collection of Biospecimens from the Inspiration4 Mission Establishes the Standards for the Space Omics and Medical Atlas (SOMA). Nat. Commun..

[25] Bojmar L (2021). Extracellular vesicle and particle isolation from human and murine cell lines, tissues, and bodily fluids. STAR Protoc..

[26] Li H, Yang H, Wang D, Zhang L, Ma T (2020). Peroxiredoxin2 (Prdx2) Reduces Oxidative Stress and Apoptosis of Myocardial Cells Induced by Acute Myocardial Infarction by Inhibiting the TLR4/Nuclear Factor kappa B (NF-κB) Signaling Pathway. Med. Sci. Monit..

[27] Chang C-W, Yang S-Y, Yang C-C, Chang C-W, Wu Y-R (2019). Plasma and Serum Alpha-Synuclein as a Biomarker of Diagnosis in Patients With Parkinson’s Disease. Front. Neurol..

[28] Lööv C, Scherzer CR, Hyman BT, Breakefield XO, Ingelsson M (2016). α-Synuclein in Extracellular Vesicles: Functional Implications and Diagnostic Opportunities. Cell. Mol. Neurobiol..

[29] Montoya MC (2002). Role of ICAM-3 in the initial interaction of T lymphocytes and APCs. Nat. Immunol..

[30] Akimova D (2017). Metabolite profiling of whole murine embryos reveals metabolic perturbations associated with maternal valproate-induced neural tube closure defects. Birth Defects Res..

[31] Kelesidis T (2015). The Role of Platelet-Activating Factor in Chronic Inflammation, Immune Activation, and Comorbidities Associated with HIV Infection. AIDS Rev..

[32] Ashraf M. Z. & Srivastav, S. Oxidized phospholipids: introduction and biological significance. in *Lipoproteins - Role in Health and Diseases* (ed. Kostner, G.) (InTech, 2012). 10.5772/50461.

[33] Klein J (2000). Membrane breakdown in acute and chronic neurodegeneration: focus on choline-containing phospholipids. J. Neural Transm..

[34] Altomare A (2021). Lipid peroxidation derived reactive carbonyl species in free and conjugated forms as an index of lipid peroxidation: limits and perspectives. Redox Biol..

[35] Wen J, Yachelini PC, Sembaj A, Manzur RE, Garg NJ (2006). Increased oxidative stress is correlated with mitochondrial dysfunction in chagasic patients. Free Radic. Biol. Med..

[36] Zhang W, Liu R, Chen Y, Wang M, Du J (2022). Crosstalk between Oxidative Stress and Exosomes. Oxid. Med. Cell. Longev..

[37] Wallace DC (2005). A mitochondrial paradigm of metabolic and degenerative diseases, aging, and cancer: a dawn for evolutionary medicine. Annu. Rev. Genet..

[38] Auten RL, Davis JM (2009). Oxygen toxicity and reactive oxygen species: the devil is in the details. Pediatr. Res..

[39] Xia C (2019). Mesenchymal stem cell-derived exosomes ameliorate intervertebral disc degeneration via anti-oxidant and anti-inflammatory effects. Free Radic. Biol. Med..

[40] Shiekh PA, Singh A, Kumar A (2020). Exosome laden oxygen releasing antioxidant and antibacterial cryogel wound dressing OxOBand alleviate diabetic and infectious wound healing. Biomaterials.

[41] Yang J (2015). Extracellular Vesicles Derived from Bone Marrow Mesenchymal Stem Cells Protect against Experimental Colitis via Attenuating Colon Inflammation, Oxidative Stress and Apoptosis. PLoS ONE.

[42] Crucian B (2016). Incidence of clinical symptoms during long-duration orbital spaceflight. Int. J. Gen. Med..

[43] Crucian B (2013). Immune system dysregulation occurs during short duration spaceflight on board the space shuttle. J. Clin. Immunol..

[44] Pierson DL, Stowe RP, Phillips TM, Lugg DJ, Mehta SK (2005). Epstein-Barr virus shedding by astronauts during space flight. Brain Behav. Immun..

[45] Rooney BV, Crucian BE, Pierson DL, Laudenslager ML, Mehta SK (2019). Herpes virus reactivation in astronauts during spaceflight and its application on earth. Front. Microbiol..

[46] Mehta SK (2014). Multiple latent viruses reactivate in astronauts during Space Shuttle missions. Brain Behav. Immun..

[47] Mehta SK, Stowe RP, Feiveson AH, Tyring SK, Pierson DL (2000). Reactivation and shedding of cytomegalovirus in astronauts during spaceflight. J. Infect. Dis..

[48] Perkins A, Nelson KJ, Parsonage D, Poole LB, Karplus PA (2015). Peroxiredoxins: guardians against oxidative stress and modulators of peroxide signaling. Trends Biochem. Sci..

[49] Nandi A, Yan L-J, Jana CK, Das N (2019). Role of Catalase in Oxidative Stress- and Age-Associated Degenerative Diseases. Oxid. Med. Cell. Longev..

[50] Kerksick C, Willoughby D (2005). The antioxidant role of glutathione and N-acetyl-cysteine supplements and exercise-induced oxidative stress. J. Int. Soc. Sports Nutr..

[51] Haskó G (2000). Inosine inhibits inflammatory cytokine production by a posttranscriptional mechanism and protects against endotoxin-induced shock. J. Immunol..

[52] Bhattacharyya S (2016). Oral Inosine Persistently Elevates Plasma antioxidant capacity in Parkinson’s disease. Mov. Disord..

[53] Jin X, Shepherd RK, Duling BR, Linden J (1997). Inosine binds to A3 adenosine receptors and stimulates mast cell degranulation. J. Clin. Invest..

[54] Marcinkiewicz J, Kontny E (2014). Taurine and inflammatory diseases. Amino Acids.

[55] Bhat R (2010). Inhibitory role for GABA in autoimmune inflammation. Proc. Natl Acad. Sci. USA.

[56] Zhang B (2021). B cell-derived GABA elicits IL-10+ macrophages to limit anti-tumour immunity. Nature.

[57] Mehta SK (2013). Reactivation of latent viruses is associated with increased plasma cytokines in astronauts. Cytokine.

[58] Escola JM (1998). Selective enrichment of tetraspan proteins on the internal vesicles of multivesicular endosomes and on exosomes secreted by human B-lymphocytes. J. Biol. Chem..

[59] Campos-Silva C (2019). High sensitivity detection of extracellular vesicles immune-captured from urine by conventional flow cytometry. Sci. Rep..

[60] Togarrati PP, Dinglasan N, Desai S, Ryan WR, Muench MO (2018). CD29 is highly expressed on epithelial, myoepithelial, and mesenchymal stromal cells of human salivary glands. Oral. Dis..

[61] Cibrián D, Sánchez-Madrid F (2017). CD69: from activation marker to metabolic gatekeeper. Eur. J. Immunol..

[62] Wang Y (2018). Pulmonary alveolar type I cell population consists of two distinct subtypes that differ in cell fate. Proc. Natl Acad. Sci. USA.

[63] Matsuda T (2001). Expression of the receptor tyrosine kinase genes, Ror1 and Ror2, during mouse development. Mech. Dev..

[64] Borcherding N, Kusner D, Liu G-H, Zhang W (2014). ROR1, an embryonic protein with an emerging role in cancer biology. Protein Cell.

[65] Legg J, Jensen UB, Broad S, Leigh I, Watt FM (2003). Role of melanoma chondroitin sulphate proteoglycan in patterning stem cells in human interfollicular epidermis. Development.

[66] de Bruyn M (2010). Melanoma-associated Chondroitin Sulfate Proteoglycan (MCSP)-targeted delivery of soluble TRAIL potently inhibits melanoma outgrowth in vitro and in vivo. Mol. Cancer.

[67] Yang H, Parkhouse RME, Wileman T (2005). Monoclonal antibodies that identify the CD3 molecules expressed specifically at the surface of porcine gammadelta-T cells. Immunology.

[68] Pols MS, Klumperman J (2009). Trafficking and function of the tetraspanin CD63. Exp. Cell Res..

[69] Fang X, Zheng P, Tang J, Liu Y (2010). CD24: from A to Z. Cell. Mol. Immunol..

[70] Heger L (2020). Subsets of cd1c+ dcs: dendritic cell versus monocyte lineage. Front. Immunol..

[71] Blase L, Daniel PT, Koretz K, Schwartz-Albiez R, Möller P (1995). The capacity of human malignant B-lymphocytes to disseminate in SCID mice is correlated with functional expression of the fibronectin receptor alpha 5 beta 1 (CD49e/CD29). Int. J. Cancer.

[72] Gupta U, Baig S, Majid A, Bell SM (2023). The neurology of space flight; How does space flight effect the human nervous system. Life Sci. Space Res. (Amst).

[73] Lee AG (2020). Spaceflight associated neuro-ocular syndrome (SANS) and the neuro-ophthalmologic effects of microgravity: a review and an update. NPJ Microgravity.

[74] Zhao S (2021). Possible role of a dual regulator of neuroinflammation and autophagy in a simulated space environment. Acta Astronaut.

[75] Mao XW (2020). Spaceflight induces oxidative damage to blood-brain barrier integrity in a mouse model. FASEB J..

[76] Bellone JA, Gifford PS, Nishiyama NC, Hartman RE, Mao XW (2016). Long-term effects of simulated microgravity and/or chronic exposure to low-dose gamma radiation on behavior and blood-brain barrier integrity. NPJ Microgravity.

[77] Zu Eulenburg P (2021). Changes in Blood Biomarkers of Brain Injury and Degeneration Following Long-Duration Spaceflight. JAMA Neurol..

[78] Amselem, S. & Eyal, S. The Blood-Brain Barrier in Space: Implications for Space Travelers and for Human Health on Earth. *Front. Drug. Deliv*. **2**, 10.3389/fddev.2022.931221 (2022).

[79] Marchi N (2003). Peripheral markers of brain damage and blood-brain barrier dysfunction. Restor. Neurol. Neurosci..

[80] Kang C (2022). Blood-brain barrier disruption as a cause of various serum neuron-specific enolase cut-off values for neurological prognosis in cardiac arrest patients. Sci. Rep..

[81] Marshall-Goebel K (2019). Assessment of jugular venous blood flow stasis and thrombosis during spaceflight. JAMA Netw. Open.

[82] Szabó LD, Keresztes P, Pallos JP, Csató E, Predmerszky T (1984). Study of nucleic acid metabolism in two astronauts. Adv. Space Res..

[83] Chen P (2016). Human metabolic responses to microgravity simulated in a 45-day 6° head-down tilt bed rest (HDBR) experiment. Anal. Methods.

[84] Kurosawa R (2021). Impact of spaceflight and artificial gravity on sulfur metabolism in mouse liver: sulfur metabolomic and transcriptomic analysis. Sci. Rep..

[85] Mao XW (2014). Biological and metabolic response in STS-135 space-flown mouse skin. Free Radic. Res..

[86] Wang Y (2016). Effect of Prolonged Simulated Microgravity on Metabolic Proteins in Rat Hippocampus: Steps toward Safe Space Travel. J. Proteome Res..

[87] Ghosh P (2016). Effects of High-LET Radiation Exposure and Hindlimb Unloading on Skeletal Muscle Resistance Artery Vasomotor Properties and Cancellous Bone Microarchitecture in Mice. Radiat. Res..

[88] Dickerson BL, Sowinski R, Kreider RB, Wu G (2023). Impacts of microgravity on amino acid metabolism during spaceflight. Exp. Biol. Med, (Maywood).

[89] Siddiqui IJ, Pervaiz N, Abbasi AA (2016). The Parkinson Disease gene SNCA: Evolutionary and structural insights with pathological implication. Sci. Rep..

[90] Chen B (2019). The impacts of simulated microgravity on rat brain depended on durations and regions. Biomed. Environ. Sci..

[91] McGregor HR (2023). Impacts of spaceflight experience on human brain structure. Sci. Rep..

[92] Berrios DC, Galazka J, Grigorev K, Gebre S, Costes SV (2021). NASA GeneLab: interfaces for the exploration of space omics data. Nucleic Acids Res.

[93] Overbey, E. G. The space omics and medical atlas and international astronaut biobank. *Nature*. 10.1038/s41586-024-07639-y (2024). 10.1038/s41586-024-07639-y PMC11357981 38862028

[94] Kim, J. et al. Single-cell multi-ome and immune profiles of the Inspiration4 crew reveal conserved, cell-type, and sex-specific responses to spaceflight. 10.1038/s41467-024-49211-2 (2023). 10.1038/s41467-024-49211-2 PMC11166952 38862516

[95] Barisic, D. et al. ARID1A orchestrates SWI/SNF-mediated sequential binding of transcription factors with ARID1A loss driving pre-memory B cell fate and lymphomagenesis. *Cancer Cell* **42**, 583–604.e11 (2024). 10.1016/j.ccell.2024.02.010 PMC11407687 38458187

[96] Love MI, Soneson C, Patro R (2018). Swimming downstream: statistical analysis of differential transcript usage following Salmon quantification. [version 3; peer review: 3 approved]. F1000Res..

[97] Khan A, Mathelier A (2017). Intervene: a tool for intersection and visualization of multiple gene or genomic region sets. BMC Bioinforma..

[98] Wang J, Vasaikar S, Shi Z, Greer M, Zhang B (2017). WebGestalt 2017: a more comprehensive, powerful, flexible and interactive gene set enrichment analysis toolkit. Nucleic Acids Res..

[99] Scott RT (2020). Advancing the integration of biosciences data sharing to further enable space exploration. Cell Rep..

[100] Houerbi N (2024). Secretome profiling reveals acute changes in oxidative stress, brain homeostasis, and coagulation following short-duration spaceflight. Zenodo.

