# Supplementary material for: Secretome profiling reveals acute changes in oxidative stress, brain homeostasis, and coagulation following short-duration spaceflight
Source: Nat Commun. 2024 Jun 11;15:4862. doi: 10.1038/s41467-024-48841-w (PMC11166969; doi:10.1038/s41467-024-48841-w)
Supplement: Supplementary file 1 — Supplementary Information [file 41467_2024_48841_MOESM1_ESM.pdf]

Supplementary Figure

Supplementary Figure 1. Volcano plots of acute changes after 3-day spaceflight.

a. Volcano plot of DAPs in plasma at R+1 vs preflight (p-adjusted < 0.05, |logFC|>1). **b.** Volcano plot of DAPs in EVPs at R+1 vs preflight (p-adjusted < 0.05, |logFC|>1).

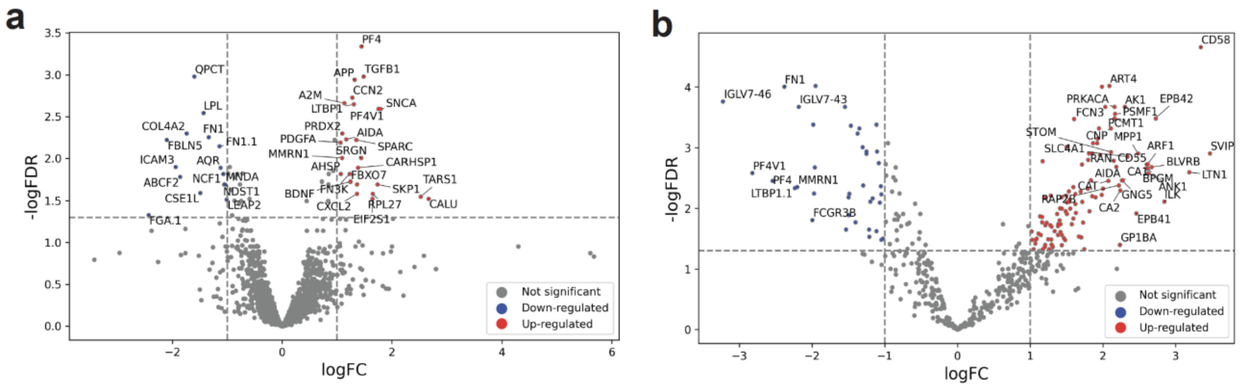

Supplementary Figure 2. Validation of PF4 change in abundance in plasma and EVPs using ELISA.

a. ELISA of PF4 in plasma isolated from cell preparation tubes (CPT). **b.** ELISA of PF4 in EVPs isolated from plasma.

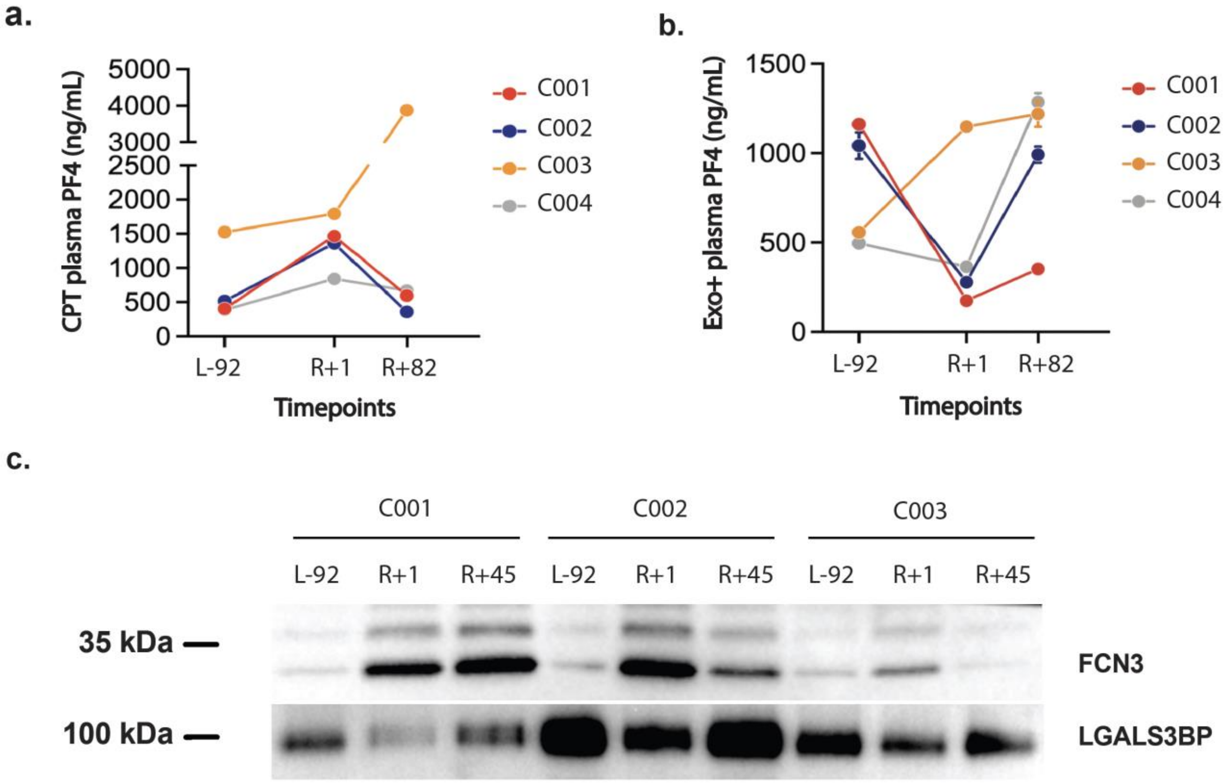

Supplementary Figure 3. Heatmap of plasma and EVP proteins involved in metabolic pathways.

Heatmaps for all I4 astronauts over time (i.e., pre-flight to multiple time points post-flight) for the individual proteins and $\log_2$(fold-change) for the mitochondrial OXPHOS, antioxidant, and custom metabolic pathways. The individual protein expression values are represented in red (upregulated proteins) and blue (downregulated proteins). The fold-change values are determined by $\log_2$(fold-change) comparing immediate and long-term postflight changes to preflight values. The p-value < 0.05 is indicated in each cell with a *. This is displayed for plasma (left heatmap) and EVP (right heatmap) samples.

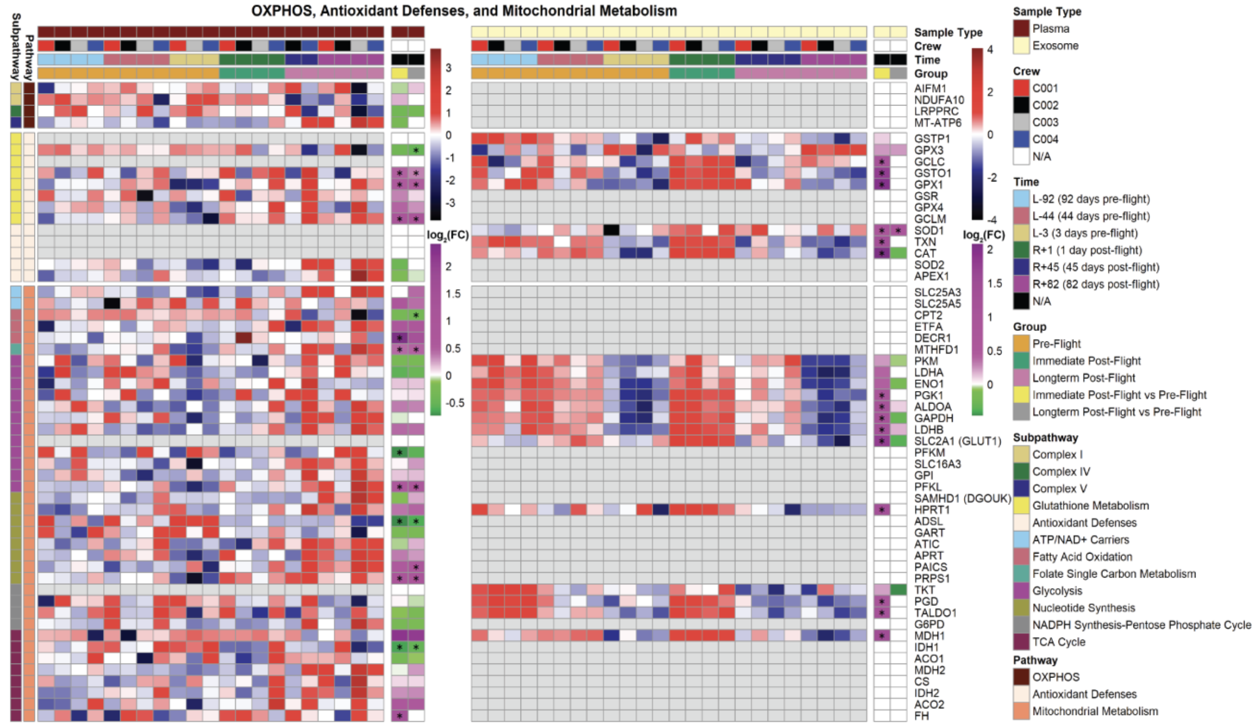

Supplementary Figure 4. Fold change (R+1/pre-flight) of the selected secretome-enriched pathways normalized score in immune cells.

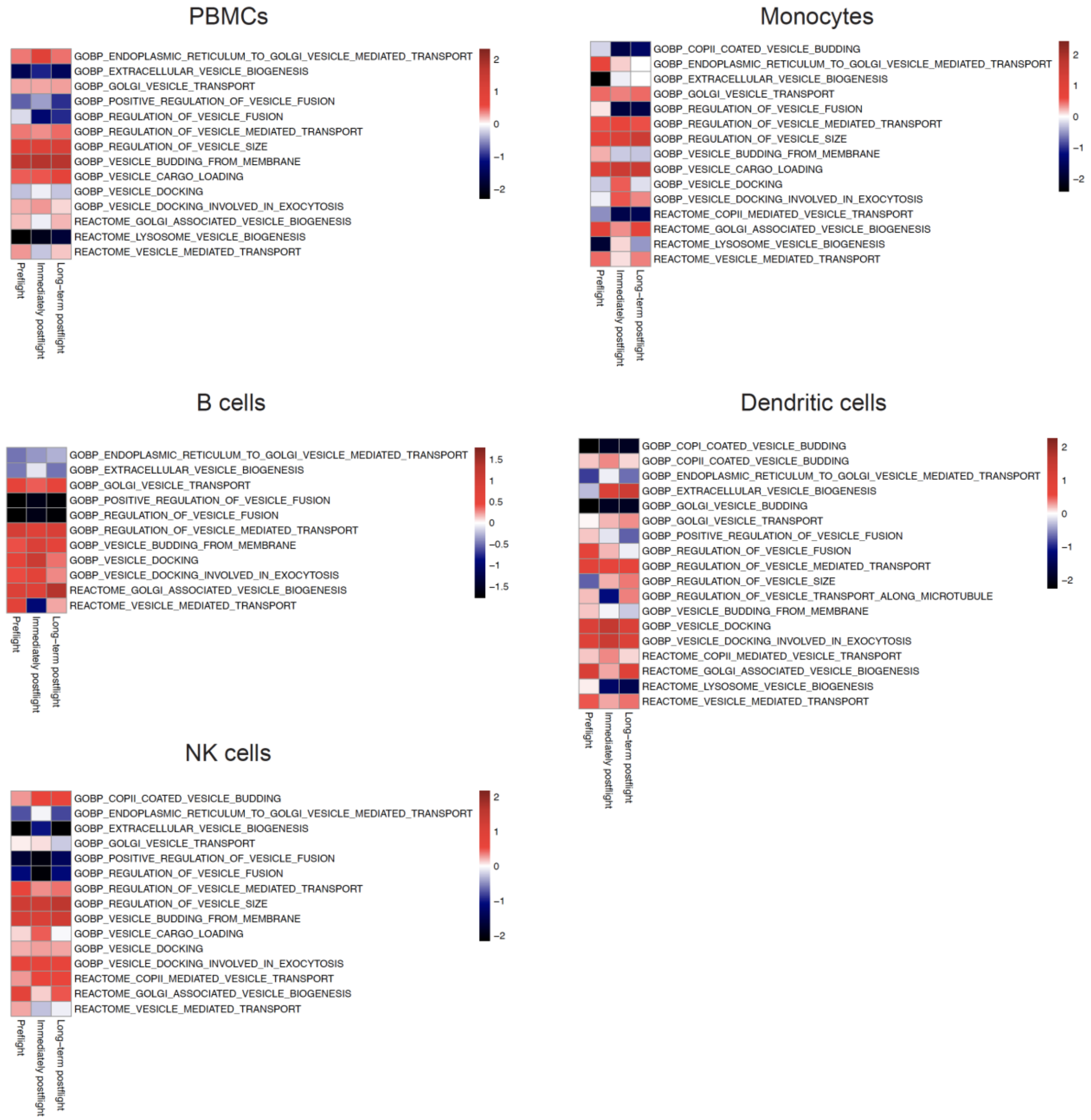

Supplementary Figure 5. Expression of MACSplex EVP surface markers in immune cells. Heatmaps represent the protein expression levels of indicated immune markers on the surface of EVPs, at the indicated timepoints, normalized to negative controls. The values were normalized by centering and dividing by the standard deviations.

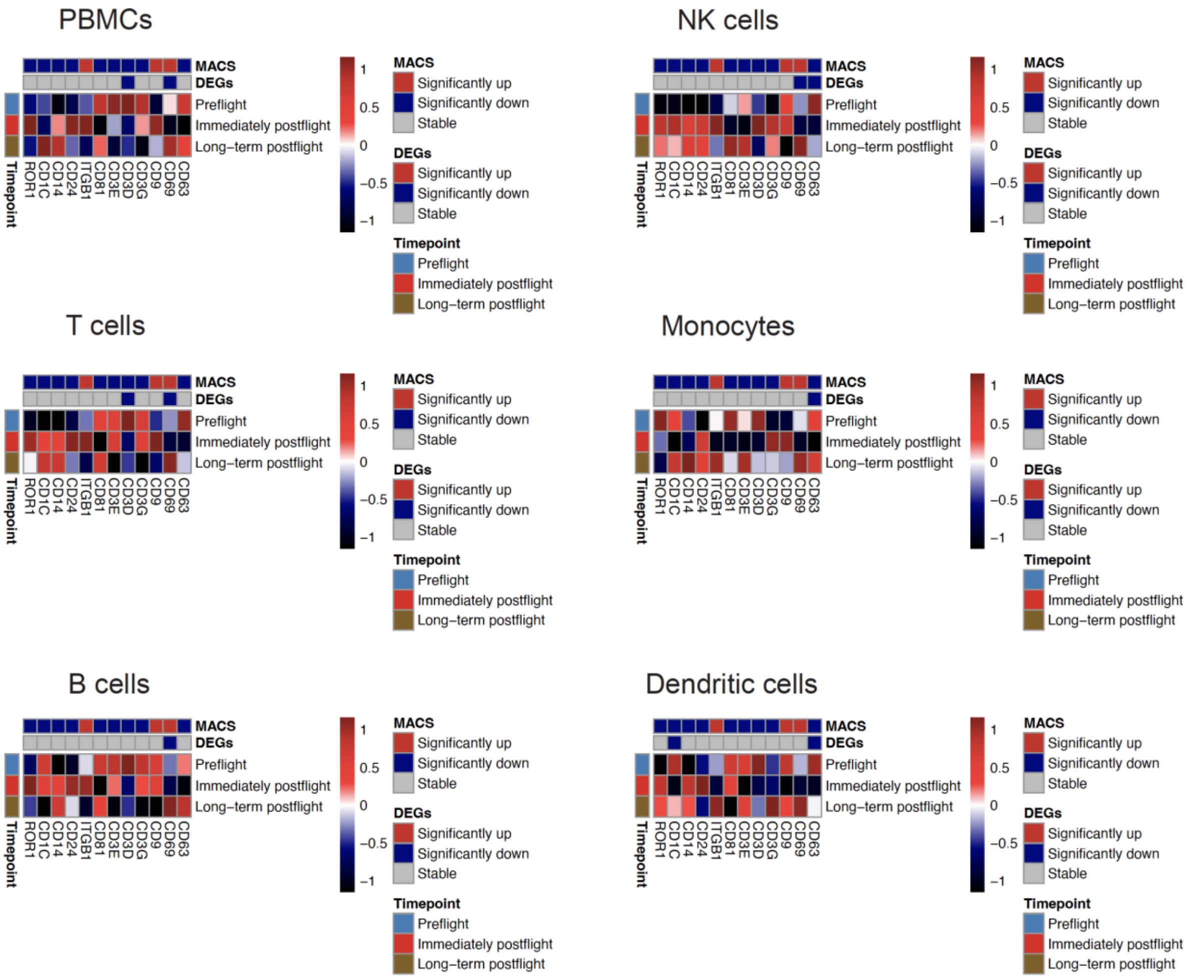

Supplementary Figure 6. Blood cells do not contribute to secretome changes after spaceflight.

a. Volcano plot of differentially expressed genes from whole blood. Genes are colored according to whether they are differentially abundant in the proteomics data. The horizontal line marks the 0.05 threshold for adjusted p-value. The vertical lines mark the |0.5| threshold for logFC. **b.** Overlap between differentially expressed genes in whole blood and differentially abundant proteins in plasma and EVPs. **c.** Whole blood gene expression, protein abundances, and PBMC gene expression for the 8 genes that are differentially expressed/abundant among whole blood and EVPs (AHSP, AK1, ANK1, BLVRB, EPB42, HBD, SELENBP1, SNCA). **d.** Overrepresentation analysis for differentially expressed/abundant genes in the whole blood gene expression and EVP proteomics data. Green boxes (left panel) display the genes/proteins included in the overrepresented set. Enrichment database types are displayed (right panel) if their FDR < 0.05.

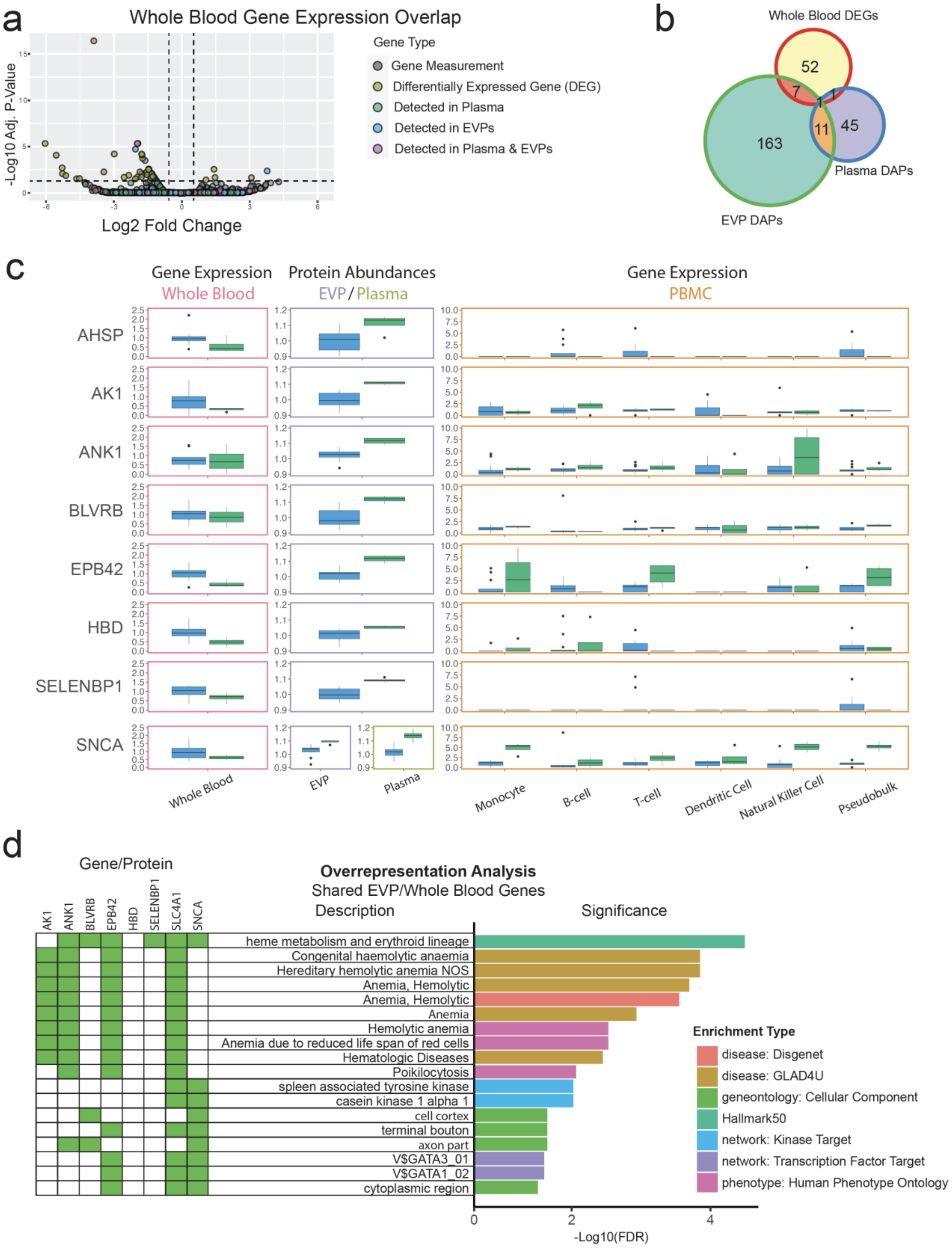
a
Whole Blood Gene Expression Overlap
-Log10 Adj. P-Value
Log2 Fold Change
Gene Type
Gene Measurement
Differentially Expressed Gene (DEG)
Detected in Plasma
Detected in EVPs
Detected in Plasma & EVPs
b
Whole Blood DEGs
52
7
1
1
45
11
163
Plasma DAPs
EVP DAPs
c
Gene Expression
Whole Blood
Protein Abundances
EVP / Plasma
Gene Expression
PBMC
AHSP
AK1
ANK1
BLVRB
EPB42
HBD
SELENBP1
SNCA
Whole Blood
EVP
Plasma
Monocyte
B-cell
T-cell
Dendritic Cell
Natural Killer Cell
Pseudobulk
d
Gene/Protein
AK1
ANK1
BLVRB
EPB42
HBD
SELENBP1
SLC4A1
SNCA
Overrepresentation Analysis
Shared EVP/Whole Blood Genes
Description
Significance
heme metabolism and erythroid lineage
Congenital haemolytic anaemia
Hereditary hemolytic anemia NOS
Anemia, Hemolytic
Anemia, Hemolytic
Anemia
Hemolytic anemia
Anemia due to reduced life span of red cells
Hematologic Diseases
Poikilocytosis
spleen associated tyrosine kinase
casein kinase 1 alpha 1
cell cortex
terminal bouton
axon part
V$GATA3_01
V$GATA1_02
cytoplasmic region
-Log10(FDR)
Enrichment Type
disease: Disgenet
disease: GLAD4U
geneontology: Cellular Component
Hallmark50
network: Kinase Target
network: Transcription Factor Target
phenotype: Human Phenotype Ontology

Supplementary table

Supplementary table 1. Data availability on NASA Open Science Data Repositories (OSDR)

**Biospecimen**	**Assay(s)**	**OSDR Identifier**
Blood Plasma	• Proteomics (Seer Proteograph)<br>• Proteomics of blood extracellular vesicles and particles<br>• Proteomic assay of blood plasma metabolome<br>• Metabolomics data<br>• Cell-free RNA	OSD-571
	• Direct RNA-seq	OSD-569
