# Supplementary material for: Secretome profiling reveals acute changes in oxidative stress, brain homeostasis, and coagulation following short-duration spaceflight
Source: Nat Commun. 2024 Jun 11;15:4862. doi: 10.1038/s41467-024-48841-w (PMC11166969; doi:10.1038/s41467-024-48841-w)
Supplement: Supplementary file 2 — Peer Review File [file 41467_2024_48841_MOESM2_ESM.pdf]

nature portfolio

Peer Review File

Secretome profiling reveals acute changes in oxidative stress, brain homeostasis, and coagulation following short-duration spaceflight

Open Access This file is licensed under a Creative Commons Attribution 4.0 International License, which permits use, sharing, adaptation, distribution and reproduction in any medium or format, as long as you give appropriate credit to the original author(s) and the source, provide a link to the Creative Commons license, and indicate if changes were made. In the cases where the authors are anonymous, such as is the case for the reports of anonymous peer reviewers, author attribution should be to 'Anonymous Referee' followed by a clear attribution to the source work. The images or other third party material in this file are included in the article's Creative Commons license, unless indicated otherwise in a credit line to the material. If material is not included in the article's Creative Commons license and your intended use is not permitted by statutory regulation or exceeds the permitted use, you will need to obtain permission directly from the copyright holder. To view a copy of this license, visit http://creativecommons.org/licenses/by/4.0/.

Editorial Note: This manuscript has been previously reviewed at another journal. This document only contains reviewer comments and rebuttal letters for versions considered at *Nature Communications*. Mentions of the other journal have been redacted.

REVIEWERS' COMMENTS

Reviewer #1 (Remarks to the Author):

This is a valuable study that applies powerful analytical techniques and data processing approaches to provide unique insight into the systematic changes induced by short-duration spaceflight. It will be an important and influential report.

The authors have addressed all of my previous comments (reviewer #3 of [redacted] submission), and I recommend acceptance of this work. Congratulations on this nice effort!
